# Experiences of medical students and doctors with dyslexia: A systematic review

**DOI:** 10.1111/medu.15615

**Published:** 2025-02-12

**Authors:** Suhail Amin Tarafdar, Noha Seoudi, Ruoyin Luo, Kalman Winston

**Affiliations:** ^1^ College of Medicine and Dentistry outreach centre of Ulster University Birmingham UK; ^2^ School of Pharmacy and Pharmaceutical Sciences Ulster University Coleraine UK; ^3^ Warwick Medical School Warwick University Coventry UK; ^4^ Institute of Continuing Education University of Cambridge Cambridge UK; ^5^ Institute of Dentistry Queen Mary University of London London; ^6^ Barts Health NHS Trust Barts Health Dental Hospital London; ^7^ Department of Public Health and Primary Care University of Cambridge Cambridge

## Abstract

**Introduction:**

Dyslexia can be defined as a neurodevelopmental learning difficulty characterised by issues with phonological awareness, affecting performance and progression within medical education. There is a need to identify how to provide effective support for medical students and postgraduate doctors with dyslexia. The aim of this systematic review was to explore the experiences of, and identify strategies for, undergraduate medical students and postgraduate medical doctors with dyslexia within existing literature.

**Methods:**

A search strategy was undertaken on databases relevant to medical education. Included studies concerned either medical students or postgraduate medical doctors with dyslexia. A quality appraisal was undertaken and narrative synthesis employed to produce a final report.

**Results:**

Thirty‐one articles were included in the final synthesis, with seven deemed high‐risk of bias. Four overarching themes were identified. There are largely negative experiences, such as stigma, reported. Furthermore, dyslexia can impact assessment performance, with reasonable adjustments effective for written examinations. Moreover, strategies employed to reduce difficulties include peer support, organisational inclusivity and interactive educational methodologies. Additionally, dyslexia impacts the career trajectory of doctors.

**Discussion:**

Training programmes should promote an inclusive environment through raised awareness and provision of reasonable adjustments. A range of potential strategies have been identified to improve the educational experiences of students with dyslexia, but these should be flexible according to individual needs. Further research is warranted within postgraduate medical training and experiences in the United States.

## Abstract 2

This review paper outlines the experiences of medical students and postgraduate doctors with dyslexia, outlining several important implications for medical training.

## 1 INTRODUCTION

Dyslexia is frequently described as a neurodevelopmental learning difficulty affecting the precision and fluency of word‐reading and spelling.^1^, ^2^ Nonetheless, this definition may be too narrow as it implies a deficit in individuals, without taking other considerations into account. Indeed, applying the ‘social model of disability’ would suggest that disability from dyslexia arises from barriers imposed on dyslexic individuals within society. Therefore, societal reformation, such as increased accessibility would overcome such disability.^3^ Furthermore, the neurodiversity approach argues that cognitive diversity within society is expected, rather than pathological, with disability arising from the interaction between the characteristics of the disabled person and their environment.^3^

Even individual experiences of dyslexia vary, it can cause issues with phonological awareness, verbal memory and processing, although strengths may include interactivity, creativity, problem‐solving and design skills.^1^, ^2^ These features can particularly manifest in demanding professions, such as medicine, with evidence that dyslexia can negatively affect the progression of, and experiences for dyslexic medical students and postgraduate doctors.^4^, ^5^, ^6^ Using an interactionist neurodiversity approach, disability from dyslexia should be addressed through reshaping society and environments, valuing the diversity of individuals, and adaptive mechanisms for individuals with dyslexia.^3^ Whilst considering this paradigm, it is important to reflect on the use of person‐first (‘person with dyslexia’) or identity‐first (‘dyslexic person’) language, because of some recommendations to utilise person‐first language, but others arguing that this may extenuate stigma.^7^ Within this paper, both forms will be used interchangeably.

The general prevalence of dyslexia is approximately 10%, although estimations vary.^2^, ^8^ Dyslexia represents around 80% of all reported learning difficulties.^9^ Five percent of UK higher education students during 2020/2021 reported a learning disability, accounting for 33% of all students with a known disability.^10^ Within UK medical schools, specific learning disability (SpLD) is the commonest declared disability, with 4.6% of medical students declaring this in 2018,^9^, ^11^ although some sources suggest higher figures, such as 18% of foundation doctors within one Trust^12^.^13^

Where dyslexia has a significant life‐long impact on a person's day‐to‐day life, it could be classed as a disability.^2^, ^10^ In these circumstances, it would be a protected characteristic under the Equality Act (2010) within England, Scotland and Wales. There are similar provisions worldwide, including the Americans with Disabilities Act (1990) and the European Union charter of Fundamental Rights, which prohibit discrimination based on disabilities within employment and other areas of life.^14^, ^15^ Therefore, medical training programmes have a duty to provide reasonable adjustments in these circumstances.^16^

Nonetheless, the literature pertaining to dyslexia within medical education is limited. One systematic review in 2015 identified strategies for clinicians with dyslexia such as approaches to medical documentation, use of adaptive technologies, increased time and improved awareness of dyslexia; however, it only included five studies,^17^ with many studies published since. Other non‐primary articles focus on strategies for supporting undergraduate medical students with learning disabilities, including dyslexia,^18^, ^19^, ^20^, ^21^ whilst one paper specifically focussed on dyslexia in postgraduate general practice (GP) training.^22^ However, numerous primary research studies have been published since, with a particular lack of systematic reviews exploring dyslexia in medical training.

Therefore, there is a need to review the literature to inform best practice for medical students and postgraduate doctors with dyslexia. The aim of this systematic review was to explore the experiences of, and identify strategies for, medical students and postgraduate doctors with dyslexia, within primary research studies. This is to inform interventions that can be utilised by medical students and postgraduate doctors with dyslexia, in addition to their educational practitioners. Furthermore, it was to identify gaps in the literature and inform other areas for research.

## 2 METHODS

In order to synthesise the available published research, a systematic review was undertaken. This methodology was selected as it is particularly rigorous for identifying recommendations to inform practice within medical education.^23^ PRISMA alongside adjunctive guidance were considered when conducting and reporting the systematic review.^24^, ^25^

### 2.1 Eligibility criteria

Studies were only included if they concerned medical students and/or postgraduate doctors with dyslexia. Only peer‐reviewed primary research studies were included to improve rigour of the data. To ensure that up‐to‐date recommendations were incorporated, studies published before 2003 were excluded. Studies unavailable in the English language were excluded.

### 2.2 Information sources

Searches were undertaken in October 2023 on PubMed, Google Scholar and the NHS Knowledge and Library Hub, which incorporated numerous scientific databases (e.g. MEDLINE, CINAHL) (these are listed in Appendix S1). These databases were chosen because of their relevance to healthcare and medical education, in addition to convenience of citation extraction. The citations of included articles and relevant reviews were also searched.

### 2.3 Search strategy

The search terms were initially selected using Boolean terms and PI(C)O methodology, ensuring that they were broad for greater capture of publications. As this review was intended to inform recommendations for both undergraduate and postgraduate medical training, search terms were included for both. The following search strategy was undertaken:

dyslexia OR dyslexic OR dyslex* OR (learning disability)

AND

(medical education) OR (medical school) OR (medical student) OR (medical trainee) OR (medical training) OR (clinical education) OR (medical student education) OR (postgraduate medical education)

AND

experiences OR perceptions OR attitudes OR views OR intervention OR strategy OR exam* OR assessment

The search strategies from different databases are outlined in Appendix S2. To improve reliability, the search was independently undertaken by two reviewers.

### 2.4 Data selection and management

Citations were imported, and the titles of papers were screened and excluded using the eligibility criteria. Thereafter, the abstracts of remaining studies were screened and irrelevant studies excluded. Finally, full‐text reviews of remaining studies were undertaken, and all papers which met the inclusion criteria were included. Additionally, 20% of citations underwent abstract and full‐text screening with a second reviewer to increase reliability.

### 2.5 Risk of bias

Each included paper underwent quality appraisal to assess the risk of bias as part of systematic review methodology. The quality appraisal tools were modified from the Critical Appraisal Skills Programme and Joanna Brigg's Institute toolkits, with individual toolkits designed for qualitative,^26^, ^27^ cross‐sectional^28^, ^29^ and cohort^30^, ^31^ studies (see Appendix S3). Where studies utilised a mixed‐methods approach, two separate quality appraisals of the same paper were undertaken for each study method. Twenty percent of included studies were quality appraised by two reviewers to check reliability of the process.

### 2.6 Data extraction, collection and synthesis

A data extraction proforma was designed (Appendix S4) to include the following components:

- Reviewer and study details;
- Methodology, including study aims, settings, design, follow‐up and duration, outcomes, analysis methods and ethical approval;
- Results of the study including experiences of dyslexia, undergraduate and postgraduate experiences, barriers and facilitators to training, assessments and other findings.

Given the diversity of included studies, with disparate methodologies and variation in quality, a narrative synthesis was employed, which drew on the principles of both textual narrative and thematic synthesis.^32^, ^33^ The former involved placing papers into homogenous groups and producing a commentary on study characteristics (context, quality and findings); the latter then followed, through coding the text of each paper to produce descriptive and analytical themes, which were used to describe the findings of the systematic review.^32^, ^33^

## 3 RESULTS

### 3.1 Study selection

**FIGURE 1:**
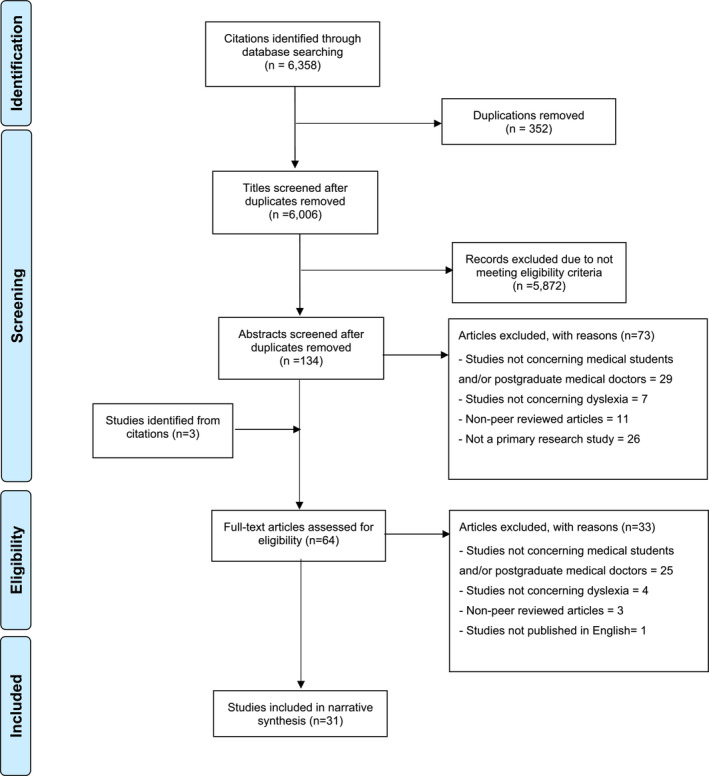
PRISMA diagram outlining the process of screening papers at different stages of the systematic review. [Color figure can be viewed at wileyonlinelibrary.com]

After removal of duplications, the search strategy identified 6006 articles. 5872 articles were excluded during title screening; 73 articles were excluded during abstract screening. Three further articles were identified after searching the citations of papers and assessed at the full‐text stage of screening. During full‐text review, 33 articles were excluded, with 31 articles included in the final narrative synthesis. Figure 1 presents the PRISMA chart outlining the process.

### 3.2 Characteristics of included studies

The characteristics of each study are outlined in Appendix S5. Thirteen papers were qualitative, nine used a cohort design, and six were cross‐sectional studies. Three papers used a mixed‐methods design: qualitative and cross‐sectional.

Twenty‐four studies were based in the UK, four in the United States, one in both United States and New Zealand, and one each in France and Australia. All studies except four had participants with dyslexia: two surveyed medical students regarding dyslexic medical students, another explored public views regarding disabilities in doctors, whilst another surveyed the views of medical teachers regarding neurodevelopmental disorders.

The majority (18 papers) only explored undergraduate education, eight concerned the experiences of postgraduate doctors, whilst five studied both. Of those exploring postgraduate training, four concerned foundation training (immediately post‐medical school), four explored GP training, one studied surgical training, and it was unclear for two studies. For studies that specifically examined a specialty training programme (GP and surgery), four out of five studied quantitative assessment‐based outcomes, whilst only one explored specialty trainee perceptions.

### 3.3 Risk of bias from included studies

A quality appraisal was undertaken of 16 qualitative, nine cohort and nine cross‐sectional designs, with three of these being mixed‐method (qualitative and cross‐sectional) and therefore appraised twice. Individual studies were considered overall high‐risk if

- Five out of 10 domains for qualitative or cohort studies were high risk or
- Four out of nine domains for cross‐sectional studies were high risk.

Appendices S6–S8 summarise the risk of bias appraisal for all study types. Altogether, seven studies were judged to be overall high risk of bias, whilst 24 were judged to be low risk.

High‐risk studies were included in the final review because of face validity of the findings, corroborated by other low‐risk studies. Moreover, three high‐risk cross‐sectional studies used a mixed‐methods approach where qualitative methodology triangulated the overall findings.^34^, ^35^, ^36^ Furthermore, some high‐risk studies provided unique insights not found in other papers, including views of the public,^35^ views of medical teachers^34^ and cognitive rehabilitation as an intervention.^37^ Therefore, the decision was made to include papers with high risk of bias.

### 3.4 Findings

There were four major themes, divided into sub‐themes (summarised in Table 1).

**TABLE 1:** A tables of the themes and sub‐themes identified from the narrative synthesis of the 31 included studies.

Theme	Sub‐themes	Summary of findings
There are largely negative experiences reported	There is stigma and poor awareness of dyslexia	Lack of awareness regarding dyslexia Protracted diagnosis in medical school or postgraduate training, often following failure Within GP training, declaration of dyslexia more likely following failure in IMGs Rise in SpLD declared at medical schools from 2002 to 2018, 4.6% medical students in 2018 Stigma amongst dyslexic medical students and doctors Negative preconceptions about what dyslexia entails Negative reactions from supervisors and peers (e.g. bullying) Reluctance to disclose dyslexia Some comfortable to disclose dyslexia because of professional responsibility and for provision of adjustments Some positive reactions reported from others (e.g. supportive colleagues or supervisors) Surveys of non‐dyslexic medical students and the public largely supportive, although some caveats to this (e.g. fairness of reasonable adjustments, suitability for role)
	There is considerable psychological burden	Considerable psychological burden reported (e.g. depression, anxiety, stress, trauma) Emotional impact (e.g. hopelessness, poor self‐esteem, guilt) Some positive emotions (e.g. relief, important part of their being, feeling proud)
Dyslexia can impact assessment performance	There are different types of reasonable adjustments for dyslexia	Within the UK, medical students with dyslexia are as likely as students without dyslexia to complete medical degree, despite lower educational performance Within the US, medical students with learning disabilities are less likely to successfully complete, graduating at later times, compared to students without learning disabilities Reasonable adjustments or accommodations for assessment include additional time, separate room, additional materials to write (pen and paper) and separate format for examination (e.g. buff paper) Preference for written exam formats, compared to computer‐based examinations Although reasonable adjustments increased confidence, sometimes personally viewed negatively, they can cause significant burden such as prolongation of difficulties or viewed negatively by others, with negative responses from some medical schools/faculties when adjustments are requested Evidence that a large proportion of medical students with SpLD/dyslexia do not utilise or request reasonable adjustments Disappointment despite passing, because of lower grades achieved
	Written assignment are challenging	Difficulties with completing written assignments because of spelling and computing information
	Reasonable adjustments are effective for written examinations	No statistically significant difference for MCQs, EMQs and SAQs, when reasonable adjustments provided (mainly extra time) for UK undergraduate and postgraduate (GP and surgery) written examinations, although a potential delay of up to 1 year for this to be seen in undergraduate medicine Candidates with SpLD are more likely to repeat written examinations for GP licensing MCQ examination Candidates declaring dyslexia after initial failure for GP licensing MCQ more likely to be IMGs Within US studies, evidence of candidates with learning disabilities performing worse in written MCQ examinations
	Simulated clinical examinations are associated with differences in attainment	Medical students with dyslexia perform worse in OSCEs concerning data‐gathering and examination skills, with one study suggesting that year 1 students with dyslexia perform worse than non‐dyslexic students for all OSCEs For GP training, candidates with dyslexia perform worse in the clinically based licensing examinations, and are more likely to sit the examinations more than once One study suggests that SpLD GP trainees are more likely to perform worse in the interpersonal skills domain of the role‐play clinical examination and in the management skills domain of real‐life recorded consultation examination For postgraduate surgical training, no evidence of differences in pass rates for OSCEs OSCEs are perceived by dyslexic medical students as more difficult than real‐life clinical practice
	Workplace‐based assessments are challenging	Within GP training, trainees with SpLD are more likely to achieve non‐standard outcomes at the ARCP, with this likelihood increasing during latter training years, suggesting difficulties with successful completion of workplace‐based assessments.
Strategies are employed to reduce difficulties related to dyslexia	Communication and organisation strategies are used for task completion	Difficulties with organisation ‐ Prioritisation of tasks, administrative tasks, keeping deadlines, poor concentration ‐ Spatial difficulties and problems telling left from right, especially in female doctors Difficulties with communication ‐ Reading difficulties: slow reading, issues with comprehension, misreading texts/charts/handwriting/numbers, computer screens difficult ‐ Issues with writing and typing, including slow speed, writing neatly and spelling, especially under pressure ‐ Difficulties writing referral letters, patient forms, patient clerking/notes, discharge summaries ‐ Issues with listening, including telephone messages, during ward rounds and educational events/lectures, or listening to others ‐ Speaking/expression difficulties, including reading out loud, presenting and handover of tasks Difficulties with prescribing ‐ Reading and completing drug charts, especially handwritten prescriptions, drug spelling and being susceptible to distractions whilst prescribing/calculating drug dosages ‐ Specific support and training for prescribing was lacking in medical school/postgraduate training, despite usual personal coping strategies for doctors/medical students with dyslexia being ineffective Strategies (organisation, communication and adaptive technologies) ‐ Additional time for reading, preparation and completion of tasks ‐ Double‐checking completed work and repetition of events ‐ Spelling aloud ‐ Prioritisation of tasks ‐ Allowing additional time and space for tasks, and ‘getting on with it’ ‐ Breaking down information: lists, bullet points, mind‐mapping, colour‐coding, AV aids, SBAR, printed patients lists, aide‐memories ‐ Font size/colour ‐ Spellcheckers ‐ Clinical templates ‐ Dictaphones/speech‐to‐text/speech recognition software ‐ Calculators ‐ Electronic resources (e.g., BNF, barcode readers, electronic flashcards, AV materials) ‐ Search engines ‐ Prescribing strategies: Seeking advice of multidisciplinary colleagues (e.g. doctors, pharmacists, dieticians, specialist nurses), engagement of cognitive functions Time consuming to do the above, risk of overpreparation, anxiety
	Peer support is important	Isolation of medical students and doctors with dyslexia, especially when falling behind Difficulties to form enduring relationships and lack of collegiality Strategies ‐ Encouraging and supportive colleagues who understand dyslexia and its challenges ‐ Asking colleagues for help, including proofreading ‐ Shadowing colleagues ‐ Peer learning and sharing information with others ‐ Dyslexia support groups ‐ Buddy to scribe during ward rounds ‐ Dyslexia workshops for medical students improve dyslexia knowledge and confidence to support dyslexia peers academically and signposting for reasonable adjustments/support
	Organisational inclusivity is important	Often lack of appropriate support from deaneries/foundation schools, NHS trusts and medical schools Sometimes annoyance and stonewalling when support requested, or support being delayed or lacking in appropriate expertise/understanding from supervisors Access to support sometimes not available until failure A lack of awareness/understanding from medical teachers regarding neurodiversity and pedagogical adaptations, although they would like training Strategies ‐ Academic and pastoral support, before and after failure, particularly if tutors experienced with supporting people with dyslexia and if continuous throughout training ‐ Prompt access to university support services ‐ Remediation should be flexible and bespoke, taking students'/trainees' personal, social, professional and mental needs into account, rather than a homogenous support programme
	Interactive educational methodologies enhance learning	Being flexible to the needs of medical students' to enhance learning Didactic, lecture‐based and self‐directed learning less effective Interactive learning more effective including problem‐based learning, tutorials, group‐work, peer‐supported learning and one‐to‐one sessions Teaching more effective with audio‐visual aids, practical/kinaesthetic tasks, diagrams, whiteboard markers/pens, logical explanations/chains and creating a backbone of knowledge Technology enhanced learning, including online learning, recording, adjusting lecture speeds, splicing software, access of slides in PPT format, audio‐visual materials (e.g. videos, flashcards), usage of apps, remote peer discussion, and access to teaching materials beforehand Some issues with online learning include lack of clinical exposure, technical issues and reduced social interaction Cognitive rehabilitation as an intervention may be effective for medical students with reading difficulties
	Empathy can be a strength	Empathy and understanding of patients' needs enhanced because of personal experiences, with improved interpersonal skills
Dyslexia impacts the career trajectory of doctors	Transition to real‐life work is challenging	Reported difficulties with transitioning from medical school to working as a junior doctor in UK studies Shadowing doctors before this transition helpful One US paper reported that experiences of residency more positive than medical school
	Dyslexia influences career choice for doctors	Tendency to choose less competitive specialties and/or those which are less likely to worsen their difficulties related to dyslexia Tendency to specialties where communication is important and where more time available: General Practice, Psychiatry, Elderly care medicine Membership of the Royal College of Physicians examination perceived as difficult Career in research perceived as difficult

*Note*: ARCP, annual review of competence progression; GP, general practice; IMGs, international medical graduates; MCQ, multiple‐choice questions; OSCEs, objective structured clinical examinations; SpLD, specific learning disability.

### 3.5 There are largely negative experiences reported

#### 3.5.1 There is stigma and poor awareness of dyslexia

Early identification of dyslexia is important for improving medical student performance and implementation of adjustments.^38^, ^39^, ^40^ Yet, there is poor awareness of dyslexia, with a protracted diagnostic process,^38^, ^41^, ^42^, ^43^, ^44^ often following academic burden and failure at medical school and/or postgraduate medical training.^11^, ^38^, ^39^, ^40^, ^41^, ^45^, ^46^, ^47^, ^48^ Masking of difficulties through compensatory measures is a reason for late identification.^38^ Specific learning disability (SpLD) is the commonest declared disability in UK medical students, affecting 4.6% in 2018.^11^ Two UK studies found that postgraduate GP trainees declaring dyslexia after failure in postgraduate medical examinations are more likely to be international medical graduates (IMGs).^6^, ^49^

There are inaccurate preconceptions of dyslexia amongst medical students, doctors and educators.^34^, ^41^, ^42^, ^43^ Studies report stigma^38^, ^39^, ^41^, ^43^, ^50^, ^51^, ^52^ and negative reactions from supervisors and peers, such as bullying and accusations of ‘faking it’.^39^, ^42^, ^43^, ^47^, ^50^, ^51^, ^52^, ^53^ There is an associated reluctance to disclose dyslexia^39^, ^40^, ^42^, ^46^, ^47^, ^50^, ^51^, ^52^, ^53^: 28% of UK medical students with SpLD did not declare this during GMC registration, in one cohort study.^11^

Nonetheless, some studies report that participants are comfortable with disclosure for reasonable adjustments/support services, or they feel it is their professional duty to disclose.^41^, ^47^, ^48^, ^53^ Also, some papers report mutual support and a lack of discrimination.^41^, ^53^ One Australian study looking at the views of the public found that 74.7% felt that people with disabilities, including learning difficulties, should be encouraged to study medicine, although potential barriers include time, expense, effort, academic capacity and longevity.^35^

#### 3.5.2 There is considerable psychological burden

There is considerable psychological burden reported for doctors and medical students with dyslexia, including guilt, poor self‐esteem, depression, anxiety, panic‐attacks, stress, post‐traumatic stress, insomnia and burnout, whilst working and training.^38^, ^39^, ^41^, ^42^, ^45^, ^48^, ^50^, ^52^ Nonetheless, some participants reported positive emotions, such as relief following the diagnosis and pride in their achievements.^38^, ^48^, ^52^, ^53^

### 3.6 Dyslexia can impact assessment performance

#### 3.6.1 There are different types of reasonable adjustments for dyslexia

UK medical students with SpLD between 2002 and 2018 had lower educational performance measures but were just as likely to complete their medical course successfully as those without SpLD, suggesting that reasonable adjustments are effective.^11^ However, two US‐based studies found that medical students with learning difficulties are less likely to successfully complete the course.^54^, ^55^

The most frequently described adjustment is additional time, but others include alternative exam formats (e.g., avoiding computer screens, buff paper), additional writing tools and separate rooms.^4^, ^5^, ^6^, ^39^, ^40^, ^42^, ^43^, ^47^, ^49^, ^51^, ^52^, ^53^, ^54^, ^55^, ^56^ Reasonable adjustments increase confidence, although negative personal feelings are sometimes reported, for example, because of prolonged burden or greater visibility of their dyslexia.^39^, ^41^, ^43^, ^51^, ^53^ Moreover, some studies indicate that not all medical students with learning difficulties request adjustments, with only 53.3% at one UK‐medical school^40^ and 25% in another US study.^55^

Furthermore, studies report negative responses from medical schools and postgraduate faculties when adjustments are requested for medical school and membership examinations.^39^, ^42^, ^52^ Adjustments are sometimes viewed negatively by non‐dyslexic candidates,^39^, ^43^, ^52^ although additional time for dyslexic medical students was considered fair by non‐dyslexic students in one UK cross‐sectional survey.^43^ Disappointment is reported even after passing, as scores are often below average, and do not always correspond to effort or formative feedback.^41^

#### 3.6.2 Written assignments are challenging

Medical students with dyslexia experience difficulties with completing written assignments because of the time taken, issues with computing information, spelling and typographic errors. Deadline extensions, proofreading and spell‐checker are some strategies employed, although these can be burdensome.^41^, ^52^, ^53^

#### 3.6.3 Reasonable adjustments are effective for written examinations

Written examinations are perceived by dyslexic medical students as friendlier than clinical assessments, although there are difficulties with long and wordy question stems.^52^, ^53^ Overall, studies suggest that reasonable adjustments are effective for removing differences in pass rates for written examinations. Whilst two US‐based studies suggest that candidates with learning difficulties are more likely to fail some written examinations,^52^, ^53^ there is no statistically significant difference in pass rates for multiple‐choice questions (MCQs), extended‐matching questions (EMQs) and short‐answer questions (SAQs) undertaken by medical students with SpLDs/dyslexia, compared to those without, when reasonable adjustments are provided within UK‐based studies, usually additional time.^4^, ^5^, ^54^, ^56^

Similarly, within postgraduate training, there is no statistically significant difference in pass rates between candidates with or without SpLD/dyslexia, for MCQs within UK general practice and surgical training.^49^, ^57^, ^58^ However, candidates declaring dyslexia after failure are more likely to be IMGs for the UK GP licensing MCQs for membership of the Royal College of General Practitioners (MRCGP).^49^

#### 3.6.4 Simulated clinical examinations are associated with differences in attainment

Simulated clinical examinations are perceived as difficult by medical students with dyslexia, more so than real‐life scenarios because of additional pressure and artificiality.^45^, ^52^ Some studies indicate that unlike written examinations, additional time is not always provided during clinical examinations.^4^ One UK study found no statistically significant difference in first‐ and second‐year medical students undertaking objective structured clinical examinations (OSCEs), with or without dyslexia.^4^ However, another UK study found that students with dyslexia perform worse in their first year of medical school, but not in later years.^5^ Furthermore, it found that first, second and third‐year medical students perform significantly worse in OSCEs concerning data‐interpretation and examination skills.^5^ A US‐based study similarly found that medical students perform worse on the clinically oriented USMLE examination.^54^

Within postgraduate settings, two UK studies found that GP trainees with SpLD/dyslexia are more likely to attempt the clinically based examination for MRCGP multiple times, and less likely to pass overall.^6^, ^57^ They performed particularly poorly for interpersonal skills, in a face‐to‐face simulated OSCE (Clinical Skills Assessment).^57^ However, for a clinically based examination comprising recorded real‐life consultations (Recorded Consultation Assessment), they performed worse in management skills but not data‐gathering or interpersonal skills.^57^ Nonetheless, there is no difference in pass rates between those with or without SpLD undertaking an OSCE‐based surgical examination to achieve membership of the Royal College of Surgeons in the UK.^58^

#### 3.6.5 Workplace‐based assessments (WPBAs) are challenging

GP trainees with SpLD in the UK are significantly more likely to receive non‐standard outcomes for their annual review of competence progression (ARCP), which slows training progression; this particularly worsens between the first and final years of training.^57^ This suggests that GP trainees with SpLD are significantly more likely to struggle with WPBAs, which are required during GP training to achieve MRCGP.^57^

### 3.7 Strategies are employed to reduce difficulties related to dyslexia

#### 3.7.1 Communication and organisation strategies are used for task completion

Doctors and medical students with dyslexia experience difficulties with completing tasks, because of issues with organisation and communication. Organisation difficulties include issues with prioritisation, multitasking, time‐keeping and sequencing of tasks, easy distractibility and poor concentration. Other difficulties relate to spatial awareness, telling left from right, and administrative tasks.^39^, ^46^, ^50^, ^59^

Communication difficulties can include reading, writing, listening and speaking. Reading issues include difficult comprehension, particularly handwriting, and issues with numbers, charts and computer screens.^38^, ^39^, ^45^, ^46^, ^50^, ^53^, ^59^ Studies also report slow writing and/or typing, particularly under pressure, in addition to spelling difficulties.^39^, ^46^, ^50^, ^53^, ^59^ These present challenges with referral letters, patient forms, note‐taking and discharge summaries.^46^, ^50^, ^53^ Listening difficulties relate to taking telephone messages, absorbing information during ward rounds and educational events, and keeping pace during lectures and/or when listening to others.^39^, ^50^, ^53^ Verbal difficulties relate to reading out loud and presenting, handover to colleagues, and information recall.^46^, ^50^ For these reasons, prescribing is particularly onerous (e.g. writing/reading prescriptions and calculations), especially as coping strategies are less effective, and specific prescribing support is lacking.^46^, ^50^, ^53^, ^59^

Spending additional time to read and write, prepare for and to complete tasks, in addition to double‐checking completed work, are reported strategies.^38^, ^50^, ^52^, ^59^ Other measures include spelling aloud, repetition, task prioritisation and allowance of extra space, as well as just ‘getting on with it’ despite difficulties.^46^, ^50^, ^59^ Other reported measures include breaking down information through lists, bullet points, mind‐mapping, colour‐coding, audio‐visual aids, using SBAR (situation, background, assessment, recommendation), printed patients lists and aide memoires.^46^, ^50^, ^59^ Adaptive technologies can also be helpful, including certain fonts, spellcheckers, clinical templates, speech recognition software or dictaphones, barcode readers, smartphones or tablets for electronic resources (e.g., BNF, medical apps, electronic flashcards, videos and audio‐visual aids), search engines/internet resources and using a calculator.^46^, ^50^, ^59^ Nonetheless, these compensatory measures can be time‐consuming and exhausting, with overpreparation and anxiety.^38^, ^45^, ^50^

Similar strategies are reported for prescribing difficulties and also include seeking the advice of colleagues within the multidisciplinary team, the engagement of cognitive functions to justify prescriptions, and electronic prescribing.^50^, ^59^

#### 3.7.2 Peer support is important

Isolation is frequently reported, particularly as medical students or postgraduate trainees with dyslexia fall behind academically or with workload.^38^, ^39^, ^41^, ^42^, ^45^, ^51^, ^52^ Participants report difficulties with forming enduring personal relationships with peers and a lack of collegiality.^41^ Therefore, peer support is a positive measure, including following examination failure.^39^, ^41^, ^45^, ^50^, ^52^, ^59^ Specific support includes encouraging colleagues who understand dyslexia, requesting help, shadowing colleagues, dyslexia support groups, peer learning, buddies to scribe during ward rounds, and asking colleagues to proofread.^46^, ^50^, ^51^, ^52^, ^53^, ^60^ Dyslexia and study skills workshops improve knowledge and confidence to support dyslexic peers.^38^, ^44^

#### 3.7.3 Organisational inclusivity is important

Support is perceived to be lacking from deaneries, foundation schools, NHS Trusts and medical schools, and even stonewalling when requested. Support, when available, can be delayed/untimely, often following failure, and/or there is a lack of expertise from supervisors.^38^, ^39^, ^41^, ^42^, ^45^, ^47^, ^48^, ^52^, ^53^ One French study found that medical teachers had limited knowledge regarding neurodevelopmental students and necessary pedagogical adaptions, such as student evaluation, awareness, referral pathways and impact on patients.^34^

Appropriate, timely, academic and pastoral support with appropriate expertise, including before failure, improve experiences.^39^, ^41^, ^45^, ^46^, ^52^, ^53^, ^59^ Moreover, remediation programmes should be flexible, taking individual, personal, social, professional and mental needs into account, rather than being one‐dimensional and generic.^45^, ^48^

#### 3.7.4 Interactive educational methodologies enhance learning

Lecture‐based didactic teaching for medical students with dyslexia is perceived as less effective than tutorials, group work and peer‐supported learning.^36^, ^41^, ^60^ Self‐directed learning is also challenging, particularly for difficult topics, such as prescribing.^53^, ^59^ Instead, interactive teaching, particularly problem‐based learning through small‐group or one‐to‐one discussion using verbal, non‐written skills to consolidate learning, enhances the experience.^41^, ^53^ Specifically beneficial techniques include audiovisual aids, practical and kinaesthetic tasks, diagrams, whiteboard markers and pens, and logical explanations.^41^

Online learning can be positive, because of reduced pressure, improved self‐control, inclusivity, and accessibility to beneficial technologies and audio–visual aids.^60^ Helpful techniques include recording of teaching and software to split lectures, prior access to learning materials and access to slides in PowerPoint format.^36^, ^53^ Nonetheless, online environments reduce clinical exposure and social interaction, whilst other drawbacks include technical and formatting issues.^60^

A further potential educational intervention is cognitive rehabilitation to improve reading speed/accuracy, which improved examination performance in one US study, albeit comprising only six medical students.^37^

#### 3.7.5 Empathy can be a strength

Perceived strengths of dyslexia are increased empathy and emotional intelligence, better communication and enhanced interpersonal skills, because of personal experiences.^38^, ^47^, ^52^, ^53^ One Australian study found that most of the public believe that doctors with disabilities are an advantage to the medical profession, as it would increase empathy.^35^

### 3.8 Dyslexia impacts the career trajectory of doctors

#### 3.8.1 Transition to real‐life work is challenging

Whilst one US‐based paper suggests that experiences of residency are positive for doctors with dyslexia,^51^ studies in the UK found difficulties with transition to working.^41^, ^45^, ^47^ Shadowing doctors prior to working is helpful.^46^

#### 3.8.2 Dyslexia influences career choice for doctors

Doctors with dyslexia tend to select less competitive specialties, in addition to those requiring less written analysis. They tend to choose careers with a high reliance on interpersonal communication skills, and/or where more time is available, including general practice, psychiatry or elderly care.^39^, ^42^, ^52^ Achieving UK membership of the Royal College of Physicians (MRCP) and research careers are perceived as difficult.^42^, ^52^, ^53^ One Australian survey found that the public feel that doctors with disabilities should carefully consider how their disability would impact their chosen specialty so that their ability to practice is not impaired.^35^

## 4 DISCUSSION

This systematic review has identified four overarching themes, divided into sub‐themes. Medical students and doctors with dyslexia largely report negative experiences, with stigma, poor awareness and considerable psychological burden. Furthermore, dyslexia can impact assessment performance, with reasonable adjustments helpful for written examinations and course completion. Nonetheless, differences in attainment can persist for simulated clinical examinations and WPBAs. Moreover, strategies reduce difficulties related to dyslexia, particularly organisational and communication adjustments to complete tasks, peer support, training programme inclusivity and interactive educational methodologies. Furthermore, dyslexia impacts the career trajectory of doctors, such as influencing career choice and affecting transition to postgraduate training. There are similar findings within higher education and allied health professions, such as nursing and physiotherapy.^61^, ^62^, ^63^, ^64^, ^65^, ^66^, ^67^, ^68^ Furthermore, there is evidence of similar difficulties in medical students and doctors with other learning differences, such as autism and ADHD,^69^, ^70^ with some of the included papers within this review also including participants with a range of disabilities.^11^, ^40^, ^47^, ^48^, ^57^

The findings highlight the importance of raising awareness of dyslexia amongst doctors, students and educators. This will promote earlier identification and support, ideally before assessment failure. Furthermore, increased awareness can reduce negative preconceptions and stigma.^71^ This may be particularly important amongst IMGs, who are more likely to be identified with dyslexia following failure in GP licensing examinations.^72^

Nonetheless, beyond raising awareness, there is a need for a cultural shift within medical education settings regarding learning differences. The review has highlighted that dyslexia is frequently viewed as a deficit, with many misconceptions about what it entails. Yet, there is evidence that dyslexia can also be associated with strengths relating to interpersonal skills, empathy and creativity. Within a social model of disability, difficulties with dyslexia arise from the expectations of society placed on dyslexic individuals.^3^ Moreover, neurodiversity should be expected, rather than all individuals conforming to neurotypicality, with one in seven individuals thought to be neurodivergent, according to one UK report,^73^ with 10% of the general population being dyslexic.^2^, ^8^, ^73^ Hence, a shift to a more inclusive and positive approach within medical training is warranted, where dyslexia and other learning differences are welcomed and programmes reviewed to ensure they are not unfairly discriminating against those with these differences. This would be in keeping with an interventionist neurodiversity approach.^3^ For example, communication should be routinely accessible to all, and organisations should foster positive attitudes amongst their staff.^63^

Peer support is important, which can be facilitated through organisational workshops to enhance understanding of dyslexia amongst all colleagues. This approach has been adopted in other higher education settings with good responses.^63^ Furthermore, basic dyslexia training should be provided for medical educators, to improve awareness of recognition and of referral pathways. Furthermore, educators should have pedagogical training on interactive educational methodologies, with evidence that interactive small‐group learning is effective for all learners.^74^ In addition, organisations should have access to educators with further specialist training to support dyslexic learners for diagnosis and recommendations for further support. Given the significant burden, training programmes should have provision for psychological support, in addition to coaching for medical students and doctors with dyslexia, to increase their familiarity with strategies to enhance learning, which can improve occupational performance within organisations.^75^ Increased inclusivity may also promote a culture of disclosure, so that reasonable adjustments are provided more readily.^76^ Moreover, provision of reasonable adjustments is a legal requirement by many institutions globally, such as the Equality Act (2010) in England, Scotland and Wales; the Americans with disabilities Act (1990) in the USA; and the European Union charter of Fundamental Rights, which prohibit discrimination based on disabilities, within employment and other activities.^14^, ^15^

**FIGURE 2:**
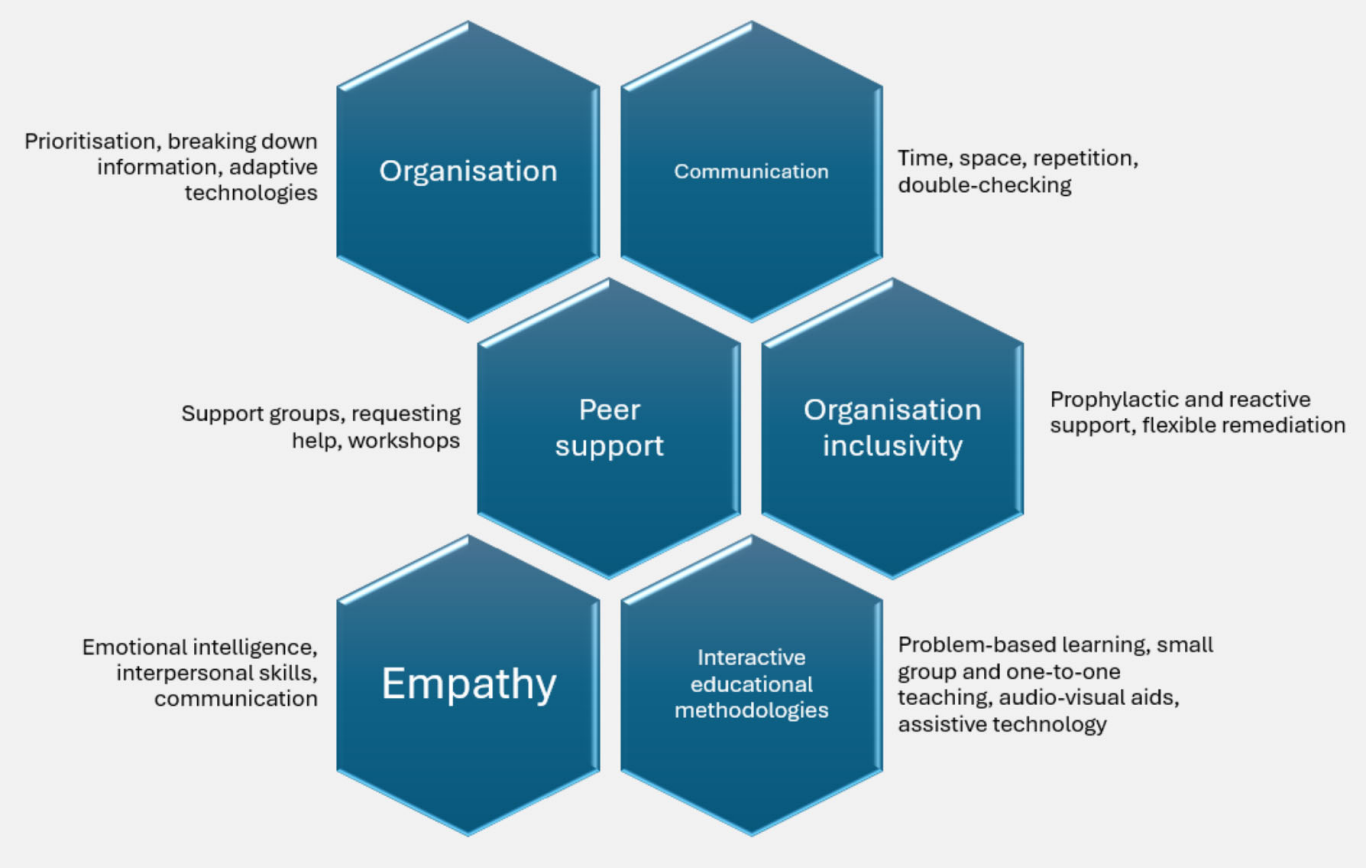
Diagram outlining strategies used to reduce difficulties with dyslexia in medical students and postgraduate doctors, with a few examples of each one. [Color figure can be viewed at wileyonlinelibrary.com]

Resources may be required for appropriate provision of reasonable adjustments in training programmes, such as universities, hospitals and/or GP surgeries. Doctors with dyslexia may be eligible for funding depending on local arrangements, such as the Access to Work grant in England.^77^ The review has identified a number of potential strategies to improve the experiences of dyslexic medical students and doctors. These are listed in Table 1, and they have been grouped into broad categories in a pictorial diagram, illustrated in Figure 2. These potential strategies might be utilised by medical students or postgraduate doctors with dyslexia, in addition to their educators. As highlighted in the review, any such strategies, support and remediation should be flexible, recognising the individuality of each person.

However, whilst the review has identified that reasonable adjustments can be effective, such as additional time removing differences in pass rates for written examinations and medical school completion, their utilisation can be associated with a significant burden on dyslexic medical students and postgraduate doctors. For example, strategies may be associated with overcompensation and prolongation of their burden. Negative reactions are also reported from organisations responsible for the incorporation of such reasonable adjustments. This should be considered by medical educators, recognising the potential psychological impact of dyslexia interventions, notwithstanding their potential effectiveness for differential attainment.

With respect to assessments, examiner training is required regarding dyslexia and reasonable adjustments, in addition to raised awareness amongst students and educators regarding their usage. Furthermore, there is a need to review the format of assessments to ensure fairness for medical students and postgraduate doctors with dyslexia. In particular, learning outcomes can be measured through a range of different assessments, including those that do not penalise neurodiverse individuals. For instance, question formatting, assessment times, and the medium for written examinations can be reviewed to make them conducive for those with dyslexia and, more broadly, neurodiversity. Moreover, there is ongoing evidence of differences in attainment related to undergraduate and postgraduate clinical‐based examinations, and workplace‐based assessments. Therefore, further research is warranted into how this could be mitigated. Thinking more broadly, assessment reviews are likely to be beneficial for other groups where differential attainment exists, such as international medical graduates and doctors with other disabilities, which may overlap with dyslexia.

### 4.1 Strengths and weaknesses

One strength of this systematic review is that a large number of studies were included, with overarching themes supported by multiple papers, using different methodologies that triangulated the findings. The methodology, based on PRISMA and adjunctive guidance, was clear and reproducible, with additional reviewers to increase reliability.

Nonetheless, seven papers were overall high risk, although the findings were corroborated by other studies. Also, not all studies specifically focussed on dyslexia; some used learning difficulties, SpLD or similar definitions. These were still included as dyslexia comprises 80% of learning difficulties.^9^ Where studies did not exclusively look at dyslexia, themes specifically pertaining to dyslexia were coded, where possible. A further limitation was that the majority of studies focussed on undergraduate experiences, and it was difficult to discern differences between undergraduate and postgraduate perceptions as studies were unclear, and/or combined data pertaining to both.

### 4.2 Gaps and insufficiencies

The study characteristics of individual studies included in the systematic review are outlined in Appendix S5. Further research is warranted regarding postgraduate specialty training within hospital and GP settings. Only one qualitative study focussed on specialty training perceptions; however, it included GP trainees who had failed to progress in training, including those without dyslexia.^48^ The remaining four studies looked at specialist training programmes through measuring quantitative assessment outcomes. Given considerable administrative and clinical differences between GP and hospital medicine, and given the specific findings of differences in attainment within simulated clinical examinations and WPBAs, more research is needed exploring dyslexia separately within general practice (family medicine) and hospital specialties. Furthermore, there is lack of data regarding the experiences of independent doctors following completion of postgraduate training. Moreover, further studies on dyslexia are warranted globally, especially the United States, given the differences reported in differential attainment for written examinations.

### 4.3 Implications for practice

Based on the findings of this systematic review, medical education programmes should adopt a more positive attitude to dyslexia, promote inclusivity and reduce stigma, through raised dyslexia awareness and appropriate provision of reasonable adjustments. Whilst reasonable adjustments are effective for removing the difference in pass rates for written examinations, there are persistent differences for simulated clinical examinations and workplace‐based assessments. Moreover, it is important to recognise the significant potential burden on dyslexic people utilising these adjustments. A range of potential strategies have been identified that can improve the educational experience of medical students and doctors with dyslexia, but these should be flexible according to individual needs. Further research is warranted looking at the experiences of specialty training, such as GP and hospital‐based training, in addition to the experiences of practice following completion of training.

## CONFLICT OF INTEREST STATEMENT

Some or all of the author(s) for this systematic review are neurodivergent themselves, in addition to having close friends, family and colleagues who are neurodivergent, including dyslexia.

## AUTHOR CONTRIBUTIONS

**Suhail Amin Tarafdar:** Conceptualization; investigation; writing–original draft; methodology; validation; visualization; writing–review and editing; formal analysis; project administration; resources. **Noha Seoudi:** Investigation; writing–review and editing; supervision; formal analysis. **Ruoyin Luo:** Supervision. **Kalman Winston:** Investigation; supervision; formal analysis; writing–review and editing.

## Supporting information


**Appendix S1:** databases searched using NHS Knowledge and Library hub.
**Appendix S2:** the specific search strategies conducted using the different databases (PubMed, NHS Knowledge and Library hub, Google scholar).
**Appendix S3:** Quality appraisal tools used for different study methodologies.
**Appendix S4:** Data extraction form used for systematic review.
**Appendix S5:** A table outlining the study characteristics and main findings for each paper included in the systematic review.
**Appendix S6:** A table highlighting the risk of bias for qualitative studies included within the systematic review.
**Appendix S7:** A table highlighting the risk of bias for the cross‐sectional studies included within the systematic review.
**Appendix S8:** A table highlighting the risk of bias for the cohort studies included within the systematic review.

## Data Availability

The additional materials are referenced in the main text and will be available in the supporting information appendix (separate document). They include the following.
