# Supplementary material for: Experiences of medical students and doctors with dyslexia: A systematic review
Source: Med Educ. 2025 Feb 12;59(8):797–811. doi: 10.1111/medu.15615 (PMC12242890; doi:10.1111/medu.15615)
Supplement: Supplementary file 1 — Appendix S1: databases searched using NHS Knowledge and Library hub. Appendix S2: the specific search strategies conducted using the different databases (PubMed, NHS Knowledge and Library hub, Google scholar). Appendix S3: Quality appraisal tools used for different study methodologies. Appendix S4: Data extraction form used for systematic review. Appendix S5: A table outlining the study characteristics and main findings for each paper included in the systematic review. Appendix S6: A table highlighting the risk of bias for qualitative studies included within the systematic review. Appendix S7: A table highlighting the risk of bias for the cross‐sectional studies included within the systematic review. Appendix S8: A table highlighting the risk of bias for the cohort studies included within the systematic review. [file MEDU-59-797-s001.docx]

**Supplementary appendix**

Supplementary file 1: databases searched using NHS Knowledge and Library hub:

- Complementary index
- Gale OneFile: Health and Medicine
- Psychology and Behavioural Sciences Collection
- Gale Health and Wellness
- MEDLINE
- Academic Search Index
- CINAHL
- APA PsychArticles
- Emerald Insight
- ScienceDirect
- Cochrane Databases of Systematic Reviews
- Directory of Open Access Journals
- Research Starters
- Journals@OVID
- Supplemental Index
- British Library EThOS
- GreenFILE
- BMJ Best Practice
- British Library Document Supply Centre Inside Serials & Conference Proceedings
- SpringerProtocols

Supplementary file 2: the specific search strategies conducted using the different databases (PubMed, NHS Knowledge and Library hub, Google scholar).

| Table 1: Search strategies conducted using various databases using Boolean logic, and the search terms above. | |
| --- | --- |
| PubMed search  (4,404 articles identified from search) | Search: **((dyslexia OR dyslexic OR dyslex* OR (learning disability)) AND ((medical education) OR (medical school) OR (medical student) OR (medical trainee) OR (medical training) OR (clinical education) OR (medical student education) OR (postgraduate medical education))) AND (experiences OR perceptions OR attitudes OR views OR intervention OR strategy OR exam* OR assessment)** Filters: **in the last 20 years**  (("dyslexia"[MeSH Terms] OR "dyslexia"[All Fields] OR "dyslexias"[All Fields] OR ("dyslexia"[MeSH Terms] OR "dyslexia"[All Fields] OR "dyslexic"[All Fields] OR "dyslexics"[All Fields]) OR "dyslex*"[All Fields] OR ("learning disabilities"[MeSH Terms] OR ("learning"[All Fields] AND "disabilities"[All Fields]) OR "learning disabilities"[All Fields] OR ("learning"[All Fields] AND "disability"[All Fields]) OR "learning disability"[All Fields])) AND ("education, medical"[MeSH Terms] OR ("education"[All Fields] AND "medical"[All Fields]) OR "medical education"[All Fields] OR ("medical"[All Fields] AND "education"[All Fields]) OR ("schools, medical"[MeSH Terms] OR ("schools"[All Fields] AND "medical"[All Fields]) OR "medical schools"[All Fields] OR ("medical"[All Fields] AND "school"[All Fields]) OR "medical school"[All Fields]) OR ("students, medical"[MeSH Terms] OR ("students"[All Fields] AND "medical"[All Fields]) OR "medical students"[All Fields] OR ("medical"[All Fields] AND "student"[All Fields]) OR "medical student"[All Fields]) OR (("medic"[All Fields] OR "medical"[All Fields] OR "medicalization"[MeSH Terms] OR "medicalization"[All Fields] OR "medicalizations"[All Fields] OR "medicalize"[All Fields] OR "medicalized"[All Fields] OR "medicalizes"[All Fields] OR "medicalizing"[All Fields] OR "medically"[All Fields] OR "medicals"[All Fields] OR "medicated"[All Fields] OR "medication s"[All Fields] OR "medics"[All Fields] OR "pharmaceutical preparations"[MeSH Terms] OR ("pharmaceutical"[All Fields] AND "preparations"[All Fields]) OR "pharmaceutical preparations"[All Fields] OR "medication"[All Fields] OR "medications"[All Fields]) AND ("trainee"[All Fields] OR "trainee s"[All Fields] OR "trainees"[All Fields])) OR (("medic"[All Fields] OR "medical"[All Fields] OR "medicalization"[MeSH Terms] OR "medicalization"[All Fields] OR "medicalizations"[All Fields] OR "medicalize"[All Fields] OR "medicalized"[All Fields] OR "medicalizes"[All Fields] OR "medicalizing"[All Fields] OR "medically"[All Fields] OR "medicals"[All Fields] OR "medicated"[All Fields] OR "medication s"[All Fields] OR "medics"[All Fields] OR "pharmaceutical preparations"[MeSH Terms] OR ("pharmaceutical"[All Fields] AND "preparations"[All Fields]) OR "pharmaceutical preparations"[All Fields] OR "medication"[All Fields] OR "medications"[All Fields]) AND ("education"[MeSH Subheading] OR "education"[All Fields] OR "training"[All Fields] OR "education"[MeSH Terms] OR "train"[All Fields] OR "train s"[All Fields] OR "trained"[All Fields] OR "training s"[All Fields] OR "trainings"[All Fields] OR "trains"[All Fields])) OR (("ambulatory care facilities"[MeSH Terms] OR ("ambulatory"[All Fields] AND "care"[All Fields] AND "facilities"[All Fields]) OR "ambulatory care facilities"[All Fields] OR "clinic"[All Fields] OR "clinic s"[All Fields] OR "clinical"[All Fields] OR "clinically"[All Fields] OR "clinicals"[All Fields] OR "clinics"[All Fields]) AND ("educability"[All Fields] OR "educable"[All Fields] OR "educates"[All Fields] OR "education"[MeSH Subheading] OR "education"[All Fields] OR "educational status"[MeSH Terms] OR ("educational"[All Fields] AND "status"[All Fields]) OR "educational status"[All Fields] OR "education"[MeSH Terms] OR "education s"[All Fields] OR "educational"[All Fields] OR "educative"[All Fields] OR "educator"[All Fields] OR "educator s"[All Fields] OR "educators"[All Fields] OR "teaching"[MeSH Terms] OR "teaching"[All Fields] OR "educate"[All Fields] OR "educated"[All Fields] OR "educating"[All Fields] OR "educations"[All Fields])) OR (("students, medical"[MeSH Terms] OR ("students"[All Fields] AND "medical"[All Fields]) OR "medical students"[All Fields] OR ("medical"[All Fields] AND "student"[All Fields]) OR "medical student"[All Fields]) AND ("educability"[All Fields] OR "educable"[All Fields] OR "educates"[All Fields] OR "education"[MeSH Subheading] OR "education"[All Fields] OR "educational status"[MeSH Terms] OR ("educational"[All Fields] AND "status"[All Fields]) OR "educational status"[All Fields] OR "education"[MeSH Terms] OR "education s"[All Fields] OR "educational"[All Fields] OR "educative"[All Fields] OR "educator"[All Fields] OR "educator s"[All Fields] OR "educators"[All Fields] OR "teaching"[MeSH Terms] OR "teaching"[All Fields] OR "educate"[All Fields] OR "educated"[All Fields] OR "educating"[All Fields] OR "educations"[All Fields])) OR (("postgraduate"[All Fields] OR "postgraduated"[All Fields] OR "postgraduates"[All Fields] OR "postgraduation"[All Fields]) AND ("education, medical"[MeSH Terms] OR ("education"[All Fields] AND "medical"[All Fields]) OR "medical education"[All Fields] OR ("medical"[All Fields] AND "education"[All Fields])))) AND ("experience"[All Fields] OR "experience s"[All Fields] OR "experiences"[All Fields] OR ("percept"[All Fields] OR "perceptibility"[All Fields] OR "perceptible"[All Fields] OR "perception"[MeSH Terms] OR "perception"[All Fields] OR "perceptions"[All Fields] OR "perceptional"[All Fields] OR "perceptive"[All Fields] OR "perceptiveness"[All Fields] OR "percepts"[All Fields]) OR ("attitude"[MeSH Terms] OR "attitude"[All Fields] OR "attitudes"[All Fields] OR "attitude s"[All Fields]) OR ("viewed"[All Fields] OR "viewing"[All Fields] OR "viewings"[All Fields] OR "views"[All Fields]) OR ("intervention s"[All Fields] OR "interventions"[All Fields] OR "interventive"[All Fields] OR "methods"[MeSH Terms] OR "methods"[All Fields] OR "intervention"[All Fields] OR "interventional"[All Fields]) OR ("strategie"[All Fields] OR "strategies"[All Fields] OR "strategy"[All Fields] OR "strategy s"[All Fields]) OR "exam*"[All Fields] OR ("assess"[All Fields] OR "assessed"[All Fields] OR "assessement"[All Fields] OR "assesses"[All Fields] OR "assessing"[All Fields] OR "assessment"[All Fields] OR "assessment s"[All Fields] OR "assessments"[All Fields]))) AND (y_10[Filter])  **Translations**  **dyslexia:** "dyslexia"[MeSH Terms] OR "dyslexia"[All Fields] OR "dyslexias"[All Fields]  **dyslexic:** "dyslexia"[MeSH Terms] OR "dyslexia"[All Fields] OR "dyslexic"[All Fields] OR "dyslexic's"[All Fields] OR "dyslexics"[All Fields]  **learning disability:** "learning disabilities"[MeSH Terms] OR ("learning"[All Fields] AND "disabilities"[All Fields]) OR "learning disabilities"[All Fields] OR ("learning"[All Fields] AND "disability"[All Fields]) OR "learning disability"[All Fields]  **medical education:** "education, medical"[MeSH Terms] OR ("education"[All Fields] AND "medical"[All Fields]) OR "medical education"[All Fields] OR ("medical"[All Fields] AND "education"[All Fields])  **medical school:** "schools, medical"[MeSH Terms] OR ("schools"[All Fields] AND "medical"[All Fields]) OR "medical schools"[All Fields] OR ("medical"[All Fields] AND "school"[All Fields]) OR "medical school"[All Fields]  **medical student:** "students, medical"[MeSH Terms] OR ("students"[All Fields] AND "medical"[All Fields]) OR "medical students"[All Fields] OR ("medical"[All Fields] AND "student"[All Fields]) OR "medical student"[All Fields]  **medical:** "medic"[All Fields] OR "medical"[All Fields] OR "medicalization"[MeSH Terms] OR "medicalization"[All Fields] OR "medicalizations"[All Fields] OR "medicalize"[All Fields] OR "medicalized"[All Fields] OR "medicalizes"[All Fields] OR "medicalizing"[All Fields] OR "medically"[All Fields] OR "medicals"[All Fields] OR "medicated"[All Fields] OR "medication's"[All Fields] OR "medics"[All Fields] OR "pharmaceutical preparations"[MeSH Terms] OR ("pharmaceutical"[All Fields] AND "preparations"[All Fields]) OR "pharmaceutical preparations"[All Fields] OR "medication"[All Fields] OR "medications"[All Fields]  **trainee:** "trainee"[All Fields] OR "trainee's"[All Fields] OR "trainees"[All Fields]  **medical:** "medic"[All Fields] OR "medical"[All Fields] OR "medicalization"[MeSH Terms] OR "medicalization"[All Fields] OR "medicalizations"[All Fields] OR "medicalize"[All Fields] OR "medicalized"[All Fields] OR "medicalizes"[All Fields] OR "medicalizing"[All Fields] OR "medically"[All Fields] OR "medicals"[All Fields] OR "medicated"[All Fields] OR "medication's"[All Fields] OR "medics"[All Fields] OR "pharmaceutical preparations"[MeSH Terms] OR ("pharmaceutical"[All Fields] AND "preparations"[All Fields]) OR "pharmaceutical preparations"[All Fields] OR "medication"[All Fields] OR "medications"[All Fields]  **training:** "education"[Subheading] OR "education"[All Fields] OR "training"[All Fields] OR "education"[MeSH Terms] OR "train"[All Fields] OR "train's"[All Fields] OR "trained"[All Fields] OR "training's"[All Fields] OR "trainings"[All Fields] OR "trains"[All Fields]  **clinical:** "ambulatory care facilities"[MeSH Terms] OR ("ambulatory"[All Fields] AND "care"[All Fields] AND "facilities"[All Fields]) OR "ambulatory care facilities"[All Fields] OR "clinic"[All Fields] OR "clinic's"[All Fields] OR "clinical"[All Fields] OR "clinically"[All Fields] OR "clinicals"[All Fields] OR "clinics"[All Fields]  **education:** "educability"[All Fields] OR "educable"[All Fields] OR "educates"[All Fields] OR "education"[Subheading] OR "education"[All Fields] OR "educational status"[MeSH Terms] OR ("educational"[All Fields] AND "status"[All Fields]) OR "educational status"[All Fields] OR "education"[MeSH Terms] OR "education's"[All Fields] OR "educational"[All Fields] OR "educative"[All Fields] OR "educator"[All Fields] OR "educator's"[All Fields] OR "educators"[All Fields] OR "teaching"[MeSH Terms] OR "teaching"[All Fields] OR "educate"[All Fields] OR "educated"[All Fields] OR "educating"[All Fields] OR "educations"[All Fields]  **medical student:** "students, medical"[MeSH Terms] OR ("students"[All Fields] AND "medical"[All Fields]) OR "medical students"[All Fields] OR ("medical"[All Fields] AND "student"[All Fields]) OR "medical student"[All Fields]  **education:** "educability"[All Fields] OR "educable"[All Fields] OR "educates"[All Fields] OR "education"[Subheading] OR "education"[All Fields] OR "educational status"[MeSH Terms] OR ("educational"[All Fields] AND "status"[All Fields]) OR "educational status"[All Fields] OR "education"[MeSH Terms] OR "education's"[All Fields] OR "educational"[All Fields] OR "educative"[All Fields] OR "educator"[All Fields] OR "educator's"[All Fields] OR "educators"[All Fields] OR "teaching"[MeSH Terms] OR "teaching"[All Fields] OR "educate"[All Fields] OR "educated"[All Fields] OR "educating"[All Fields] OR "educations"[All Fields]  **postgraduate:** "postgraduate"[All Fields] OR "postgraduated"[All Fields] OR "postgraduates"[All Fields] OR "postgraduation"[All Fields]  **medical education:** "education, medical"[MeSH Terms] OR ("education"[All Fields] AND "medical"[All Fields]) OR "medical education"[All Fields] OR ("medical"[All Fields] AND "education"[All Fields])  **experiences:** "experience"[All Fields] OR "experience's"[All Fields] OR "experiences"[All Fields]  **perceptions:** "percept"[All Fields] OR "perceptibility"[All Fields] OR "perceptible"[All Fields] OR "perception"[MeSH Terms] OR "perception"[All Fields] OR "perceptions"[All Fields] OR "perceptional"[All Fields] OR "perceptive"[All Fields] OR "perceptiveness"[All Fields] OR "percepts"[All Fields]  **attitudes:** "attitude"[MeSH Terms] OR "attitude"[All Fields] OR "attitudes"[All Fields] OR "attitude's"[All Fields]  **views:** "viewed"[All Fields] OR "viewing"[All Fields] OR "viewings"[All Fields] OR "views"[All Fields]  **intervention:** "intervention's"[All Fields] OR "interventions"[All Fields] OR "interventive"[All Fields] OR "methods"[MeSH Terms] OR "methods"[All Fields] OR "intervention"[All Fields] OR "interventional"[All Fields]  **strategy:** "strategie"[All Fields] OR "strategies"[All Fields] OR "strategy"[All Fields] OR "strategy's"[All Fields]  **assessment:** "assess"[All Fields] OR "assessed"[All Fields] OR "assessement"[All Fields] OR "assesses"[All Fields] OR "assessing"[All Fields] OR "assessment"[All Fields] OR "assessment's"[All Fields] OR "assessments"[All Fields] |
| NHS Knowledge and Library Hub  (1,390 articles identified from search) | ( dyslexia OR dyslexic OR dyslex* OR (learning disability) ) AND ( (medical education) OR (medical school) OR (medical student) OR (medical trainee) OR (medical training) OR (clinical education) OR (medical student education) OR (postgraduate medical education) ) AND ( experiences OR perceptions OR attitudes OR views OR intervention OR strategy OR exam* OR assessment ) |
| Google Scholar  (564 articles identified from search) | Intitle:dyslexia OR intitle:dyslexic OR intitle:dyslex* OR intitle:‘‘learning disability’’ AND ‘‘medical education’’ OR ‘‘medical school’’ OR ‘‘medical student’’ OR ‘‘medical trainee’’ OR ‘‘medical training’’ OR ‘‘clinical education’’ OR ‘‘medical student education’’ OR ‘‘postgraduate medical education’’ AND experiences OR perceptions OR attitudes OR views OR intervention OR strategy OR exam* OR assessment |

Supplementary file 3: Quality appraisal tools used for different study methodologies.

| ***Quality Appraisal Tool***  ***(Adapted from Joanna Briggs Institute and CASP toolkit)*** | | |
| --- | --- | --- |
| ***Qualitative Papers*** | | |
| ***Risk of Bias Item*** | ***Risk of Bias Levels*** | ***Comments*** |
| Was there a clear statement of the aims and/or objectives of the research? | Low Risk: The aims of the research were clear, including its importance and relevance |  |
|  | High Risk: The aims/objectives of the research were not clear |  |
| Is qualitative methodology appropriate for the research question? | Low Risk: The qualitative methodology is appropriate, as it is an exploratory study |  |
|  | High Risk: The qualitative methodology is not appropriate |  |
| Was the research design appropriate to address the aims of the research? | Low Risk: The research methodology is clearly described, justified and appropriate |  |
|  | High Risk: The research methodology is not appropriate |  |
| Was the recruitment strategy appropriate to the aims of the research? | Low Risk: A clear description of how and why participants were selected with that methodology, which is appropriate for the research aims |  |
|  | High Risk: The recruitment strategy is unclear and/or inappropriate |  |
| Was the data collected in a way that addressed the research issue? | Low Risk: Data collection settings and method was clear and appropriate |  |
|  | High Risk: Data collection method is not appropriate or clear |  |
| Has the relationship between researcher and participants been adequately considered? | Low Risk: There is evidence of reflexivity and the voice of the participants are represented |  |
|  | High Risk: There is no evidence of reflexivity and/or the voice of the participants do not appear to be represented |  |
| Have ethical issues been taken into consideration? | Low Risk: Ethical issues have been considered and consent sought appropriately |  |
|  | High Risk: Ethical issues have not been clearly considered |  |
| Was the data analysis sufficiently rigorous? | Low Risk: The analysis is clearly described and the results clearly explained |  |
|  | High Risk: The analysis is not clearly described and the results are not clear |  |
| Are the findings clear and flow from the data analysis? | Low Risk: The findings are clear with consideration of their credibility, such as trustworthiness or triangulation |  |
|  | High Risk: The findings are unclear and there is no consideration of trustworthiness |  |
| How valuable is the research? | Low Risk: The relevance and contribution of the research is considered, with comparisons made to the literature |  |
|  | High Risk: The relevance and contribution is not clear |  |
| ***Quality Appraisal Tool***  ***(Adapted from Joanna Briggs Institute and CASP toolkit)*** | | |
| ***Cohort Papers*** | | |
| ***Risk of Bias Item*** | ***Risk of Bias Levels*** | ***Comments*** |
| Did the study address a clearly focused issue? | Low Risk: The population, variables and outcomes are clear |  |
|  | High Risk: The issue is not focused or clear |  |
| Was the cohort recruited in an acceptable way? | Low Risk: The cohort population was clearly defined and participants were appropriately included |  |
|  | High Risk: The cohort was not clearly defined or included appropriately |  |
| Was the exposure accurately measured to minimise bias? | Low Risk: Measurements were objective, validated and the exposure was the same for the different arms in the study |  |
|  | High Risk: Measurements were not objective, validated and/or the exposure was not the same for the different cohort groups |  |
| Was the outcome accurately measured to minimise bias? | Low Risk: The measurements were objective, reliable and uniform across all groups included in the study |  |
|  | High Risk: The measurements were objective, reliable and uniform across all groups included in the study |  |
| Have the authors identified all important confounding factors and taken this into account for the analysis? | Low Risk: Confounders are considered and an appropriate analysis undertaken to account for these |  |
|  | High Risk: Confounders are not considered and/or an appropriate analysis has not been undertaken to account for these |  |
| Was the follow-up of subjects complete and the duration of follow-up appropriate? | Low Risk: There was sufficient follow-up of the participants for the study aims and research question, with consideration of participants lost to follow-up |  |
|  | High Risk: The follow-up of participants was inadequate to draw conclusions |  |
| What are the results of the study? | Low Risk: The appropriate outcomes are considered, calculated and presented appropriately (e.g., risk ratio) |  |
|  | High Risk: The appropriate outcomes are not considered, calculated and/or presented appropriately |  |
| How precise are the results? | Low Risk: The precision is calculated and presented, demonstrating significance/non-significance (e.g., confidence interval ranges) |  |
|  | High Risk: The precision is not calculated and/or presented |  |
| How plausible are the results? | Low Risk: The results appear to be plausible and an intelligent comparison can be made to existing knowledge regarding the subject matter (if available) |  |
|  | High Risk: The results do not appear to be plausible |  |
| How generalisable are the results? | Low Risk: The results can be generalised to the population of interest |  |
|  | High Risk: The results cannot be generalised to the population of interest |  |
| ***Quality Appraisal Tool***  ***(Adapted from Joanna Briggs Institute)*** | | |
| ***Cross-sectional/Observational Papers*** | | |
| ***Risk of Bias Item*** | ***Risk of Bias Levels*** | ***Comments*** |
| Were the criteria for selection in the sample clearly defined? | Low Risk: The inclusion/exclusion criteria are clear and appropriate |  |
|  | High Risk: The selection criteria are unclear and/or inappropriate |  |
| Were the study subjects and the setting described in detail? | Low Risk: The study setting and subject details are clearly described |  |
|  | High Risk: The study setting and/or subject details are not clearly described |  |
| Was the exposure measured in a valid and reliable way? | Low Risk: The exposure is measured in a valid or reliable way |  |
|  | High Risk: The exposure is not measured in a valid and/or reliable way |  |
| Were objective, standard criteria used for measurement of the issue? | Low Risk: The outcome measures are appropriate and clear |  |
|  | High Risk: The outcome measures are not appropriate or clear |  |
| Were confounding factors identified? | Low Risk: Appropriate confounders are considered in the design and analysis |  |
|  | High Risk: Appropriate confounders are not considered |  |
| Were strategies to deal with confounding factors stated? | Low Risk: Appropriate strategies are undertaken to account for confounders in the study |  |
|  | High Risk: Appropriate strategies are not undertaken for confounders in the study |  |
| Were the outcomes measured in a valid and reliable way? | Low Risk: Outcomes are measured using objective measurements |  |
|  | High Risk: Outcomes are not measured objectively |  |
| Was appropriate statistical analysis used? | Low Risk: Data is presented appropriately, with sufficient data analysis that is appropriate for the information collected |  |
|  | High Risk: Data presentation and/or analysis is inappropriate |  |
| How generalisable are the results? | Low Risk: The results can be generalised to the population of interest |  |
|  | High Risk: The results cannot be generalised to the population of interest |  |

Supplementary file 4: Data extraction form used for systematic review.

| **Reviewer Details** |  | |
| --- | --- | --- |
| **Date** |  | |
| ***Does the paper meet the inclusion criteria? If no, explain further*** |  | |
| ***Does the paper meet the exclusion criteria? If yes, explain further*** |  | |
| ***Study Details*** | | |
| **Study Title** |  | |
| **Author(s)** |  | |
| **Year** |  | |
| **Journal** |  | |
| ***Study Method*** | | |
| **Study Aims** | |  |
| **Setting** | |  |
| **Study Design** | |  |
| **Follow-up or Study Duration** | |  |
| **Subject Characteristics** | |  |
| **Outcomes** | |  |
| **Outcome Measurements** | |  |
| **Ethical Approval** | |  |
| **Method of Data Analysis** | |  |
| ***Results (Possible Domains) for Thematic Analysis*** | | |
| **Experiences of dyslexia diagnosis** | |  |
| **Medical school (undergraduate experiences)** | |  |
| **Postgraduate training experiences: GP / Hospital** | |  |
| **Barriers for training: undergraduate / postgraduate** | |  |
| **Facilitators for training:**  **Undergraduate / postgraduate** | |  |
| **Dyslexia and assessments** | |  |
| **Other domains** | |  |
| **General Practice Training Relevance** | |  |
| **Authors’ Comments** | |  |
| **Reviewer Comments** | |  |

| **Supplementary file 5: A table outlining the study characteristics and main findings for each paper included in the systematic review.** | | | | | | |
| --- | --- | --- | --- | --- | --- | --- |
| **Study ID** | **Design** | **Aims** | **Undergraduate or postgraduate** | **Country** | **Settings and participants** | **Main findings related to dyslexia** |
| Laatsch (2009) | Cross-sectional study | Examine the characteristics of individuals referred for treatment after multiple failures on the United States Medical Licensing Examinations (USMLE) Step 1 or 2 and to evaluate treatment effectiveness of cognitive. rehabilitation (CR), with a focus on reading fluency and accuracy | Undergraduate (progressing to postgraduate) licensing examination | USA | 6 medical students in the USA, who had failed the USMLE Step 1 or 2 | All participants improved in a quantitative measure of reading speed and accuracy following CR. Furthermore, 5 out of 6 students subsequently passed their next USMLE Step examination, despite previously failing this. However, given the small numbers, further research is warranted. |
| Ricketts, Brice and Coombes (2010) | Cohort study | Determine whether the adjustments for dyslexia and other specific learning difficulties (SpLDs) during MCQs are adequate and whether gender/ethnicity contribute to variation in scores | Undergraduate | UK | 900 Hull York medical students across 8 MCQ tests, covering the performance of 5 year-groups in 2006-2007, and 5 year-groups in 2007-2008 | Students with SpLDs who are given adjustments during MCQs such as additional time, perform the same as those without dyslexia. There were no differences found according to age or ethnicity. |
| McKendree and Snowling (2011) | Cohort study | Compare the results between students with and without dyslexia on all summative assessments within one UK medical school | Undergraduate | UK | Medical student end-of-year examination scores for year 1 and year 2 of the medicine degree were analysed, involving 4 cohorts of students who entered between 2004 to 2007 | Students with dyslexia were provided with additional time for written examinations, but not OSCEs. There was no significant difference between those with and those without dyslexia in the end-of-year assessments for years 1 and 2, even when controlling for age, gender and ethnicity. |
| Gibson and Leinster (2011) | Cohort study | Determine the impact of dyslexia on examination performance in medical school and whether adjustments to these assessments make a difference to the outcomes | Undergraduate | UK | 5 cohorts of medical students, starting between 2004-2008, on the MBBS programme at the University of East Anglia were analysed, undertaking end-of-year assessments comprising MCQs, SAQs and OSCEs | Students with dyslexia did less well in year 1 for all assessments, but this was not present for later years of the MBBS programme. Students with dyslexia performed better when allowed extra time for written assessments, compared to those without concessions. Students with dyslexia tended to do worse in OSCE stations concerning examination skills and data interpretation, within years 1, 2 and 3 of the MBBS. The initial poorer performance in year 1 may related to delayed adjustment to medical school and/or concessions being implemented. |
| Shrewsbury and Wiskin (2013) | Mixed methods: cross-sectional & qualitative studies | Understand the experiences of medical students with SpLDs | Undergraduate | USA and New Zealand | Medical students in the USA or New Zealand completed a survey, with a total of 325 responses. 27 students reported exam failure, and 5 of these declared an SpLD (3 of whom reported dyslexia) | It is difficult to formulate conclusions specifically related to dyslexia and/or SpLD as the majority of responses were from those who did not declare these. Medical students preferred a variety of methods to be incorporated within teaching and mentoring, (e.g., buddy system to support learners, support different learning styles, blended learning). |
| Newlands, Shrewsbury and Robson (2015) | Qualitative study | Develop an understanding of the challenges and support requirements for doctors with dyslexia in their first year of practice | Postgraduate | UK | 7 doctors undertaking their foundation year 1 placement in Scotland, between 5 to 10 months into their employment | There was a reluctance of FY1 doctors to disclose their dyslexia diagnosis to colleagues and/or supervisors. They reported difficulties with communication (oral, reading and writing), time management (e.g., prioritisation), anxiety and issues with tasks (e.g., handover, prescribing, ward round, learning environment). However, coping strategies included safety-netting (e.g., repeat-checking), organisation (e.g., lists, team-working, planning, extra time), overlearning, humour and technology (e.g., spellchecker, dictaphone). |
| Patel *et al* (2015) | Qualitative study | Investigate the perceptions and experiences of medical students who have failed final-year examinations | Undergraduate | UK | 3 medical students in 2 UK medical schools, who had failed their final-year examinations and had been asked to repeat the year for remediation. One of these students had been formally diagnosed with dyslexia. | The student with dyslexia felt isolated and reported difficulties with assimilation of information and lengthy times for reading, with too much depth involved for revision. Failure was associated with poor self-esteem, anxiety, depression and guilt. Drawing on the support of others (e.g., counsellors, personal tutors, peers and group study) was helpful. Despite individual circumstances for each student, remediation interventions were one-dimensional that only focused on passing written exams. Therefore, generic remediation programmes may not meet the needs of students experiencing failure, which may include personal, social and mental health factors. |
| Searcy *et al* (2015). | Cohort study | Determine whether MCAT scores obtained with standard versus additional administration time affects the acceptance to medical school, or the future medical student performance | Undergraduate | USA | 2 time-intervals were studied: applicants to US medical schools for 2011-2012, including those with standard time (n=133,962) versus extra time (n=435), and of students who matriculated in US medical schools from 2000-2004, with MCAT scores obtained with standard (n=76,262), versus extra time (n=449). | Extra time and/or non-standard breaks were allowed for eligible students, with the majority due to mental impairments (learning or psychiatric disabilities, ADD, ADHD etc). There was no significant difference in acceptance rates to medical school in those who had or did not have additional time. Students with extra time for MCAT were significantly less likely to pass USMLE Step examinations first-time and graduated from medical school at significantly lower rates at different times, even when controlling for MCAT scores and undergraduate point averages. The findings might be explained due to learning environments and support systems not being conducive to the needs of those requiring additional time for MCAT admission tests. |
| Romberg, Shaywitz and Shaywitz (2016) | Qualitative study | Examine the dilemmas faced by a medical student with dyslexia, particularly related to disclosure of their diagnosis to receive accommodations | Undergraduate | USA | The paper utilised quotations and the experiences of several medical students, although the exact number is unclear | Dyslexia in medical students is associated with stigma, concerns about disclosure, negative reactions and lack of understanding from supervisors, peers and colleagues (including in residency programmes). However, supportive supervisors can facilitate their education and experiences. |
| Shaw, Anderson and Grant (2016) | Qualitative study | Provide a detailed account of the impact of dyslexia in a UK medical student, to help students and academic support staff, to outline improvements within curricula, and to promote further research in this area | Undergraduate | UK | The account is an autoethnography of one final-year medical student in the UK with dyslexia | Dyslexia in medical students can be associated with a personal negative emotional reaction, a lack of understanding from self and others, and stigma. It is also associated with isolation, both academically and socially. Reduced performance was experienced in examinations and written assessments (e.g., essays). Coping strategies included collaboration with others, including supportive supervisors, peers and colleagues. In addition, some learning methods were conducive to learning with dyslexia (e.g., 1-to-1, 'backbone' of knowledge, practical skills, novel learning methods). |
| Shaw and Anderson (2017) | Qualitative study | Explore the lived experiences of medical students and doctors with dyslexia | Undergraduate and Postgraduate | UK | 8 junior doctors, comprising 7 foundation year (FY1 and FY2) doctors, and 1 GP trainee | Participants reported stigma, bullying and negative reactions from others, with concerns about disclosure of dyslexia. There was a perceived lack of support and understanding of dyslexia from medical schools and deaneries, even when requesting accommodation for assessment. Furthermore, they experienced isolation and issues related to workload and continual assessment. Trainees wanted to pursue less competitive and/or more communication-based specialties. |
| Locke *et al* (2017) | Qualitative study | Explore the impact of dyslexia for doctors on clinical practice and coping strategies used by them to mitigate these | Postgraduate | UK | 14 doctors with dyslexia were involved, in addition to 5 informants from the Professional Support Unit, which supports junior doctors with personal/educational needs | Diagnosis tended to be in medical school, but doctors were reluctant to disclose their diagnosis to employers. Doctors experienced difficulties related to communication (written, reading, verbal), organisation and time management, poor short-term memory, negative attitudes from colleagues and non-enabling adjustments. Strategies to cope concerned these difficulties, such as templates, spell-checkers, Dictaphones, breaking-up text, font size, audio-visual aids, assistive technologies, adequate time and space, supportive colleagues and shadowing. |
| Tso (2018) | Qualitative study | Explore the experiences of disabled medical students on the graduate-entry medicine programme at Warwick medical school | Undergraduate (graduate-entry medicine) | UK | 8 medical students at Warwick medical school who had disclosed a disability. The range of disabilities reported included dyslexia, dyspraxia, partial sightedness, deafness and physical disability | Diagnosis of dyslexia was in medical school, after examination failure. Participants expressed concerns about disability disclosure, although some felt that it was their duty as part of professionalism. They found medicine more challenging than their initial degree, with issues related to inadequate adjustments for disability and negative reactions from others. However, disabilities fostered their empathy towards patients. |
| Asghar *et al* (2018). | Cross-sectional study | Compare the performance of GP trainees undertaking the MRCGP AKT (written MCQ) exam with a declaration of dyslexia, compared to all other GP trainee candidates without a declaration of dyslexia | Postgraduate | UK | Data was collected for candidates taking the AKT on one or more occasions between 2010 and 2015, with a total of 14 AKT examinations involving 14,801 candidates, of which 379 (2.6%) declared dyslexia | The pass rate for candidates who declared dyslexia was 83.6%, compared to 95.0%, but this difference was not significant after adjusting for covariates. The majority of these candidates who declared dyslexia, were granted extra time for the exam. Candidates declaring dyslexia after an initial AKT failure were more likely to be IMGs. Furthermore, a significantly greater proportion of candidates declared dyslexia if they were male, aged 30 years or above, or had multiple exam attempts. Candidates who were female, under 30 years of age and with a PMQ within the UK, were more significantly more likely to pass the AKT. However, male candidates, of Black or Asian ethnicity, and who had failed the AKT previously, were significantly more likely to fail the AKT. |
| Shaw and Anderson (2018) | Qualitative study | Explore the experiences of medical students with dyslexia, to elicit meaning and understanding from their lived experiences | Undergraduate experiences (postgraduate participants) | UK | 8 junior doctors recruited from a UK foundation school, who had studied in 5 different medical schools. | Some participants reported stigmatisation/feelings of inadequacy and felt that medical school was a highly competitive environment, feeling out of place. Due to dyslexia, some respondents planned to pursue less competitive specialties, or those which were slower-paced and allowed double-checking and confirmation (e.g., writing prescriptions). They also reported isolation, self-consciousness and hopelessness/helplessness. There was reluctance to disclose the diagnosis and negative responses from others, such as bullying and stonewalling. However, there were experiences of pride in achievement despite dyslexia, pastoral and peer support, and development of strengths related to dyslexia (e.g., interpersonal skills, working harder). While MCQs and EMQs were viewed as dyslexia-friendly, essays and OSCEs were perceived as more difficult, with negative experiences sometimes reported when reasonable adjustments were requested for assessments. |
| Asghar, Williams and Denney (2019) | Cross-sectional study | Compare the performance of GP trainees undertaking the MRCGP CSA (OSCE) exam with a declaration of dyslexia, compared to GP trainees without a declaration of dyslexia | Postgraduate | UK | Data was collected for candidates taking the CSA between 2010 and 2017, with 20,879 candidates, of which 598 (2.9%) declared dyslexia | GP trainees who declared dyslexia were significantly more likely to attempt the CSA more than once, and 85.3% of dyslexia candidates passed overall, compared to 96.4% of those who did not declare dyslexia. Passing candidates were more likely to be female and have taken the CSA once, compared to those who did not declare dyslexia. Candidates who declared dyslexia following an initial failure were more likely to be an IMG or belong to a minority ethnic group. |
| Mogensen and Hu (2019) | Mixed methods: cross-sectional & qualitative studies | Investigate the views of the community/public concerning doctors and medical students with disabilities | Undergraduate and postgraduate | Australia | 207 respondents from the community completed the survey, based in the Greater Western Sydney region in New South Wales Australia | 92.3% of the respondents knew someone with a disability (long standing disability, illness, mental health condition or learning difficulty). The majority (79.7%) felt that people with disabilities should be accepted into medical schools and that this could even be advantageous for the medical profession (81.4%). They commented that fair selection is important, in addition to monitoring and support from medical schools. Furthermore, life experiences could promote empathy and medical role modelling. Nonetheless, participants felt that doctors had a responsibility to monitor their own health and ability to perform, as well as consider their career specialty choice with respect to their disability (e.g., an impairment impeding their ability). Other concerns raised related to the time, expense and effort incurred, as well as the likely academic capacity, functionality in stressful environments, and real-life capacity for doctors/medical students with disabilities to work under high-pressure even after finishing medical school. |
| Ali *et al* (2019) | Qualitative study | Explore the impact of dyslexia on the educational experiences of undergraduate students in medicine, dentistry, dental therapy and biomedical sciences | Undergraduate | UK | 15 undergraduate students with dyslexia, including 5 from medicine, 4 from dentistry, 3 from dental therapy, and 3 from biomedical sciences | Most participants did not have issues with disclosing their dyslexia, and felt a diagnosis was beneficial in terms of explaining their academic issues and provision of adjustments. Participants reported issues with communication (verbal and written, particularly prescribing and spelling) and self-directed learning. There were concerns that employers might prefer someone without a disability due to their dyslexia, and they were less keen to pursue a career in research. Facilitators included accessing university support services, advanced preparation, support from peers, use of software/gadgets (e.g., recording), PBL as it involved more discussion, practical skills, online lectures in PPT form, and supervisors experienced with dyslexia. Adjustments for assessments were deemed helpful, although they found long vignettes during MCQs frustrating. Furthermore, written assignments were considered difficult, and participants used proof reading and technology to mitigate this. There was a preference for written tests, compared to computer tests and participants felt that markers/assessors needed awareness of dyslexia so that appropriate adjustments could be made. |
| Gray and Burr (2020) | Cohort study | Investigate whether the timing of the SpLD diagnosis and implementation of modified assignment provision (MAP) affects the performance in applied medical knowledge MCQ assessments | Undergraduate | UK | 108 students diagnosed with an SpLD and who received MAP were compared to 1,960 students without MAP, over 5 years of the medical programme at Peninsula medical school, with students between 2002 and 2016 studied | A significantly higher number of medical students were diagnosed in their first year of study. There was a significant increase in SpLD diagnosis from the third year of the programme, likely related to increasing pressures of the curriculum. Students who received MAP increased their performance in the applied knowledge MCQs, although this effect was not statistically significant until 1 year after implementation of MAP. |
| Anderson and Shaw (2020) | Cross-sectional study | Quantify the extent of bullying and lack of support for medical students and doctors with dyslexia (following on from a qualitative study) | Undergraduate and postgraduate (postgraduate participants) | UK | 77 junior doctors recruited from 5 foundation schools in the South of England | The majority (53%) were diagnosed with dyslexia at university; those who were diagnosed before medical school felt that an earlier diagnosis was helpful (88%). There were negative reactions (personally and from others) related to dyslexia, including bullying, shame, psychological issues and isolation. There was a particular lack of support from foundation schools/deaneries. Participants reported problems with communication, attainment, and with the transition from medical school to working as a junior doctor. Supports included adjustments for assessment (e.g., extra time, separate room, writing things down), IT hardware and peer support. Support at medical school was perceived to be largely supportive, compared to postgraduate training. |
| Hennessy, Shaw and Anderson (2020) | Cross-sectional study | Investigate the knowledge of non-dyslexic medical students regarding dyslexia and medical students with dyslexia (MSWD), as well as the attitudes and behaviours of non-dyslexic medical students towards MSWD, in addition to their views on reasonable adjustments | Undergraduate | UK | 123 medical students without a diagnosis of dyslexia, spanning all 5 years of the programme at a UK medical school | Most (94%) knew someone with dyslexia and felt they knew what it entailed (97%). 29% felt that MSWD might be 'faking it', while 44% felt that MSWD might be exaggerating/exploiting it unfairly. 50% had witnessed a negative comment regarding dyslexia, compared 35% with a positive one. Some participants felt that medical school was not appropriate for MSWD. 95% felt that the provision of extra times for assessment for MSWD was appropriate, while a minority (9%) felt that additional supports (e.g., computer software) were unfair. There was a significant association between medical school year group and the percentage who believed that extra provision was unfair, with more students in later years believing it to be unfair. |
| Winter, Norman and Patel (2021) | Qualitative study | Understand the factors that trainees perceive to have contributed to their failure to progress within GP training | Postgraduate | UK | 23 GP trainees in the UK were interviewed, who had failed to progress in their training, some of whom were diagnosed with dyslexia | There were 3 global categories: personal factors (e.g., personal beliefs and well-being), professional factors (e.g., training and development) and social factors (e.g., family and work-life balance). Trainees who received a diagnosis of dyslexia often suspected that something was wrong, and it was a relief for some to have an answer. Seeking support was generally straightforward, although not always timely and trainees did not always exhibit stereotypical features of dyslexia. There was a lack of knowledge regarding appropriate learning strategies for postgraduate training and a lack of emotional/social support for trainees. There were issues with heavy workload, poor motivation, lack of family time and psychological issues. There was a perception that trainees' needs were not understood to facilitate appropriate, bespoke packages of remediation that addressed their individual needs. |
| Magnin, Ryff and Moulin (2021) | Mixed methods: cross-sectional & qualitative studies | Describe medical teachers' opinions of students with neurodevelopmental disorders and the management of these students | Postgraduate and undergraduate (postgraduate participants) | France | 175 medical teachers at the University of Lyon who were undertaking a postgraduate pedagogic degree for grade advancements (n=27), or who were part of a medical teacher faculty (n=148) | Many medical teachers reported that they encountered students with neurodevelopmental disorders (dyspraxia 33%; dyslexia 46%; ASD 68%; ADHD 75%), although they generally felt that their knowledge regarding these and available pedagogic adaptations, were limited. Teachers reported concerns surrounding ethical issues and they felt that the impact of neurodevelopment disorders for the student and teacher was important. They were interested in receiving advanced training for neurodevelopmental disorders in students. |
| Meeks *et al* (2022) | Cohort study | Assess the performance and trajectory of medical students with disabilities (SWDs) | Undergraduate | USA | 171 medical students with disabilities (SWD) and 341 controls across 11 MD-granting medical schools in the USA; most of the SWDs had cognitive/learning disabilities (118/171, 69%) | Medical students with physical/sensory disabilities had similar times to graduation, Step 1 scores and match on first-attempt. However, medical students with cognitive/learning disabilities had significantly lower Step 1 scores, were less likely to graduate on time, and match on first attempt. Accommodated students with cognitive/learning disabilities, however, had higher Step 1 scores, compared to those without accommodations: although not statistically significant, the increase does suggest a measurable impact of accommodations, and accommodations eliminated more than half the score difference between SWDs and controls. Furthermore, only 25% of the students had accommodations for Step 1. |
| Shaw, Hennessy and Anderson (2022) | Qualitative study | Explore the learning experiences of medical students with dyslexia during the COVID-19 pandemic, including e-learning, self-study, clinical experiences and emotional impact | Undergraduate | UK | 5 medical students, spanning years 1-3 of medical school; 3 were pre-clinical and 2 were within clinical years | Participants had positive learning experiences during the COVID-19 pandemic due to taking control (e.g., self-study, self-timetabling, own space and time, flexibility), accessibility of technologies (e.g., changing speed of lecture, online question bank, electronic flashcard, videos), peer learning (e.g., using Facetime), access to one's own space and time. There was a sense of reduced pressure and stress, and they perceived that their medical school had gone to great lengths to ensure effective distance-learning and inclusivity, in addition to increased kindness and acceptance from peers/others related to the pandemic. However, some found that home-working ate into personal time. Nonetheless, participants felt that traditional face-to-face lectures were less useful. Nonetheless, disadvantages of online learning related to reduced social/peer interaction, lack of clinical exposure, concerns about cheating in clinical assessments as practical face-to-face assessments were cancelled, technical/technological issues, and technical issues related to online assessment (e.g., small text boxes for SAQs). |
| Shaw, Okorie and Anderson (2022) | Qualitative study | Explore the experiences of a doctor with dyslexia regarding prescribing and prescribing education, drawing on their experiences as medical student and as a postgraduate doctor | Undergraduate and postgraduate | UK | The account is an autoethnography of one medical student in the UK with dyslexia, drawing on their experiences as a medical student and as a doctor | Learning how to prescribe relied on self-directed and passive learning. There was a lack of specific support for learning prescribing skills at medical school or during postgraduate training. Usual personal adaptations for dyslexia were ineffective for prescribing skills. Particular challenges with prescribing as a junior doctor related to calculation of correct doses, the lengthy time taken to prescribe, difficulties reading handwritten prescriptions and multiple distraction from others when trying to prescribe (e.g., queries from nurses, allied staff, medical students). Adaptations included support from colleagues/peers (e.g., pharmacists, dieticians), justification of each drug (e.g., related to investigations), an open supportive working environment, electronic prescribing, colour coding, and double-checking prescriptions. |
| Murphy, Dowell and Smith (2022) | Cohort study | Examine the factors associated with a declaration of disability by medical students and doctors, and the association of declared disability with academic performance | Undergraduate and postgraduate | UK | All students (n=135,930) starting at UK medical schools between 2002 and 2018, using the UK Medical Education database, a repository of data relating to training of medical students and doctors | Specific learning disability (SpLD) was the commonest declared disability (3.5%); however, the number declaring SpLD increased from 1.4% in 2002, to 4.6% in 2018. Factors predicting recording of SpLD on entry to medical school included attendance at a fee-paying school, graduate status, and participation of local areas quintile. First-year medical students were less likely to declare SpLD if they were non-White ethnicity, female, or a from a low-index of social deprivation. 28% of those with SpLD did not declare this at GMC registration. Students diagnosed with SpLD had significantly lower education performance measures, but were as likely to complete the course, compared to those without SpLD. However, the data did not measure adjustments for assessments. |
| Botan *et al* (2022) | Cohort study | Investigate the performance of doctors with SpLDs across the range of licensing assessments for GP training, to achieve Membership of the Royal College of General Practitioners (MRCGP) | Postgraduate | UK | 2,070 doctors were followed up from taking the MSRA examination from 2016 to 2017, of which 214 (10.34%) declared an SpLD; they undertook assessments during GP training including AKT, CSA, RCA and WPBAs, with outcomes measured until 2021 | Candidates with an SpLD did have lower pass rates for all assessments, but this was only statistically significant for the CSA. Furthermore, candidates with an SpLD were more likely to have more than one AKT (MCQ), CSA (clinical OSCE), and/or RCA (recorded clinical assessment) attempt. There was evidence that candidates with SpLD were significantly more likely to experience issues with WPBAs: only 54.67% of SpLD candidates had a standard ARCP outcome, compared to 82.6% of those without SpLD. The percentage of SpLD candidates receiving non-standard ARCP outcomes increased with every new evaluation (usually annually), particularly between the second/third evaluation. Having an SpLD was also a significant predictor of poor performance in RCA clinical management and CSA interpersonal skills domains. |
| Ellis *et al* (2022) | Cohort study | Assess the impact of disabilities on the performance on the Intercollegiate examination for Membership of the Royal College of Surgeons (MRCS) | Postgraduate | UK | All UK MRCS candidates attempting part A (n=9600) and part B (n=4560) between 2007 to 2017, with linked disability data from the UK Medical Education database | Candidates with disabilities had significantly lower first-time pass-rates for part A (knowledge MCQ), compared to those without disabilities. However, when adjusting for prior academic performance and sociodemographic factors, there was no statistical significance. Furthermore, there was no statistical difference in the performance of candidates with disability for part B (OSCE). When adjusting to type of disability, including specific learning difficulties (SpLD), there were also no statistical differences for either examination. |
| Cornwell and Shaw (2023) | Qualitative study | Explore potential barriers that could prevent or deter those with undiagnosed (or unrecognised) dyslexia from entering medical schools in the UK, and how those barriers may relate to the larger societal issues surrounding widening access to medicine. | Undergraduate | UK | The account is an autoethnography of a 4^th^ year medical student in the UK with dyslexia, drawing on their experiences on applying for medical school. | There is a significant emotional toll from unrecognized dyslexia, which impacted performance on meeting the requirements for entry into medical school, including pre-medical school assessments. Lack of support is associated with isolation and socio-cultural factors can impact the success of individuals. Appropriate, early support can improve success in medical school applications. Masking is a particular barrier identified to diagnosis of dyslexia, although high-achieving schools makes masking more difficult (and therefore identifying dyslexia). Dyslexia can be associated with strengths that would benefit the medical profession, including empathy, emotional intelligence and good communication. |
| Osei-Junior and Vorona (2023) | Cross-sectional study | Understand medical students’ perceptions of dyslexia and confidence with supporting fellow peers with dyslexia before and after a workshop on dyslexia. | Undergraduate | UK | 36 first-year medical students in the UK took part in an intervention awareness group, with a pre and post-evaluation form completed | Medical students knowledge and beliefs regarding dyslexia is limited. Before the dyslexia awareness workshop, the mean score on knowledge and beliefs about dyslexia was 15.22. Following the workshop, this improved to 24.03 (p<0.001). Furthermore, the confidence questionnaire indicates an improvement in confidence of medical student peers, with supporting dyslexia peers academically or signposting to reasonable adjustments and further support. |

| **Supplementary file 6: A table highlighting the risk of bias for qualitative studies included within the systematic review** | | | | | | | | | | |
| --- | --- | --- | --- | --- | --- | --- | --- | --- | --- | --- |
| **Study ID** | **Was there a clear statement of the aims and/or objectives of the research?** | **Is qualitative methodology appropriate for the research question?** | **Was the research design appropriate to address the aims of the research?** | **Was the recruitment strategy appropriate to the aims of the research?** | **Was the data collected in a way that addressed the research issue?** | **Has the relationship between researcher and participants been adequately considered?** | **Have ethical issues been taken into consideration?** | **Was the data analysis sufficiently rigorous?** | **Are the findings clear and flow from the data analysis?** | **How valuable is the research?** |
| *Shrewsbury and Wiskin (2013) | High risk | Low risk | High risk | Low risk | High risk | High risk | Low risk | High risk | High risk | High risk |
| Newlands, Shrewsbury and Robson (2015) | Low risk | Low risk | Low risk | Low risk | Low risk | High risk | Low risk | Low risk | Low risk | Low risk |
| Patel *et al* (2015) | Low risk | Low risk | Low risk | Low risk | Low risk | Low risk | Low risk | Low risk | Low risk | Low risk |
| Romberg, Shaywitz and Shaywitz (2016) | High risk | High risk | High risk | High risk | High risk | High risk | High risk | High risk | High risk | High risk |
| Shaw, Anderson and Grant (2016) | Low risk | Low risk | Low risk | Low risk | Low risk | Low risk | Low risk | Low risk | Low risk | Low risk |
| Shaw and Anderson (2017) | Low risk | Low risk | Low risk | Low risk | Low risk | Low risk | High risk | Low risk | Low risk | Low risk |
| Rachel Locke *et al* (2017) | Low risk | Low risk | Low risk | Low risk | Low risk | High risk | Low risk | Low risk | Low risk | Low risk |
| Tso (2018) | Low risk | Low risk | Low risk | Low risk | Low risk | Low risk | Low risk | Low risk | Low risk | Low risk |
| Shaw and Anderson (2018) | Low risk | Low risk | Low risk | Low risk | Low risk | Low risk | Low risk | Low risk | Low risk | Low risk |
| *Mogensen and Hu (2019) | Low risk | Low risk | Low risk | Low risk | Low risk | High risk | Low risk | Low risk | Low risk | Low risk |
| Ali *et al* (2019) | Low risk | Low risk | Low risk | Low risk | Low risk | High risk | Low risk | Low risk | Low risk | Low risk |
| Winter, Norman and Patel (2021) | Low risk | Low risk | Low risk | Low risk | High risk | High risk | Low risk | Low risk | Low risk | Low risk |
| *Magnin, Ryff and Moulin (2021) | Low risk | Low risk | High risk | Low risk | Low risk | High risk | High risk | Low risk | High risk | Low risk |
| Shaw, Hennessy and Anderson (2022) | Low risk | Low risk | Low risk | Low risk | Low risk | Low risk | Low risk | Low risk | Low risk | Low risk |
| Shaw, Okorie and Anderson (2022) | Low risk | Low risk | Low risk | Low risk | Low risk | Low risk | Low risk | Low risk | Low risk | Low risk |
| Cornwell and Shaw (2023) | Low risk | Low risk | Low risk | Low risk | Low risk | Low risk | Low risk | Low risk | Low risk | Low risk |
| * Study was a mixed-methods design which incorporated both qualitative and cross-sectional methodologies. Therefore, a risk of bias assessment has been undertaken for both study methodologies. | | | | | | | | | | |

| **Supplementary file 7: A table highlighting the risk of bias for the cross-sectional studies included within the systematic review** | | | | | | | | | |
| --- | --- | --- | --- | --- | --- | --- | --- | --- | --- |
| **Authors** | **Were the criteria for selection in the sample clearly defined?** | **Were the study subjects and the setting described in detail?** | **Was the exposure measured in a valid and reliable way?** | **Were objective, standard criteria used for measurement of the issue?** | **Were confounding factors identified?** | **Were strategies to deal with confounding factors stated?** | **Were the outcomes measured in a valid and reliable way?** | **Was appropriate statistical analysis used?** | **How generalisable are the results?** |
| Laatsch (2009) | Low risk | Low risk | Low risk | Low risk | High risk | High risk | Low risk | High risk | High risk |
| *Shrewsbury and Wiskin (2013) | High risk | High risk | High risk | High risk | High risk | High risk | High risk | High risk | High risk |
| Asghar *et al* (2018) | Low risk | Low risk | Low risk | Low risk | Low risk | Low risk | Low risk | Low risk | Low risk |
| Asghar, Williams and Denney (2019) | Low risk | Low risk | Low risk | Low risk | Low risk | Low risk | Low risk | Low risk | Low risk |
| *Mogensen and Hu (2019) | Low risk | Low risk | Low risk | High risk | High risk | High risk | Low risk | High risk | High risk |
| Anderson and Shaw (2020) | High risk | High risk | Low risk | Low risk | High risk | High risk | High risk | Low risk | Low risk |
| Hennessy, Shaw and Anderson (2020) | Low risk | Low risk | Low risk | High risk | Low risk | Low risk | High risk | High risk | Low risk |
| *Magnin, Ryff and Moulin (2021). | High risk | High risk | Low risk | Low risk | High risk | High risk | High risk | High risk | High risk |
| Osei-Junior and Vorona (2023) | Low risk | High risk | Low risk | Low risk | High risk | High risk | Low risk | High risk | Low risk |
| * Study was a mixed-methods design which incorporated both qualitative and cross-sectional methodologies. Therefore, a risk of bias assessment has been undertaken for both study types. | | | | | | | | | |

| **Supplementary file 8: A table highlighting the risk of bias for the cohort studies included within the systematic review** | | | | | | | | | | |
| --- | --- | --- | --- | --- | --- | --- | --- | --- | --- | --- |
| **Study ID** | **Did the study address a clearly focused issue?** | **Was the cohort recruited in an acceptable way?** | **Was the exposure accurately measured to minimise bias?** | **Was the outcome accurately measured to minimise bias?** | **Have the authors identified all important confounding factors and taken this into account for the analysis?** | **Was the follow-up of subjects complete and the duration of follow-up appropriate?** | **What are the results of the study?** | **How precise are the results?** | **How plausible are the results?** | **How generalisable are the results?** |
| Ricketts, Brice and Coombes (2010) | Low risk | Low risk | Low risk | Low risk | Low risk | Low risk | Low risk | Low risk | Low risk | Low risk |
| McKendree and Snowling (2011) | Low risk | Low risk | Low risk | Low risk | Low risk | Low risk | Low risk | Low risk | Low risk | Low risk |
| Gibson and Leinster (2011) | Low risk | Low risk | Low risk | Low risk | High risk | Low risk | Low risk | Low risk | Low risk | Low risk |
| Searcy *et al* (2015) | Low risk | Low risk | Low risk | Low risk | Low risk | Low risk | Low risk | Low risk | Low risk | Low risk |
| Gray and Burr (2020) | Low risk | Low risk | Low risk | Low risk | High risk | Low risk | Low risk | Low risk | Low risk | Low risk |
| Meeks *et al* (2022) | Low risk | Low risk | Low risk | Low risk | High risk | Low risk | Low risk | Low risk | Low risk | Low risk |
| Murphy, Dowell and Smith (2022) | Low risk | Low risk | Low risk | Low risk | Low risk | Low risk | Low risk | Low risk | Low risk | Low risk |
| Botan *et al* (2022) | Low risk | Low risk | Low risk | Low risk | Low risk | Low risk | Low risk | Low risk | Low risk | Low risk |
| Ellis *et al* (2022) | Low risk | Low risk | Low risk | Low risk | Low risk | Low risk | Low risk | Low risk | Low risk | Low risk |
